# Adaptive Phoneme State Learning Architecture for Enhanced Speech Recognition Using Backpropagation Neural Network and Hidden Markov Model

**DOI:** 10.12688/f1000research.177414.1

**Published:** 2026-03-02

**Authors:** Rashmi Siddalingappa, Deepa S, Margaret Savitha, Kalpana P, Priya Stella Mary I, Shivanand Gornale, Lakshmi B A, Kefeng Li, Khang Wen Goh

**Affiliations:** 1 Computer and Data Science, York St John University, London, England, E14 2BA, UK; 2 Christ University, Bengaluru, Karnataka, India; 3 Department of Computer Science, Rani Channamma University, Belagavi, Karnataka, India; 4 UST Global, Bangalore, Karnatake, India; 5 Macao Polytechnic University, Macau, Macao; 6 INTI International University & Colleges, Nilai, Negeri Sembilan, Malaysia

**Keywords:** acoustic modeling, back propagation neural networks, hidden markov model, speech recognition, voice activity detection

## Abstract

Speech remains a primary mode of human communication; however, automated speech recognition (ASR) systems face challenges from accent variability, temporal fluctuations, noise, and data privacy concerns. This paper proposes an enhanced ASR architecture incorporating an Adaptive Phoneme State Learning (APSL) algorithm with a Backpropagation Neural Network (BPNN) and Hidden Markov Model (HMM). APSL dynamically adjusts HMM state probabilities using phoneme confidence scores derived from the BPNN, thereby improving phoneme transition modeling and alignment. The multi-stage ASR pipeline includes noise reduction, speech-pause detection, and feature extraction via framing and windowing. APSL’s adaptive mechanism reduces ambiguities in phoneme transitions, resulting in a more accurate speech-to-text conversion. A comparative evaluation framework assesses the baseline HMM, standalone BPNN, and integrated APSL-BPNN-HMM model. Experiments were conducted using a custom-built dataset of 2000 audio files alongside five benchmark corpora: BNC, ANC, COCA, Buckeye, and Emu. Key evaluation metrics—recall, precision, F-score, and Word Error Rate (WER)—demonstrate that the APSL-enhanced model significantly outperforms baseline systems, achieving 95.7% recall, 92.95% precision, 94.53% F-score, and 96% overall accuracy. Notably, APSL-BPNN-HMM consistently yielded the lowest WER across all datasets, validating its effectiveness. This work highlights the benefits of adaptive learning in probabilistic frameworks for achieving robust and accurate speech recognition.

## 1. Introduction

Speech is a dynamic cascade of thoughts produced by articulating utterances in natural language. The visual representation of language is called ‘graphemes,’ while the sound representation is called ‘phonemes.’ In linguistics, the study of phonemes encompasses “Phonetics” and “Phonology.” Phonetics examines the physical properties of speech sounds, including their production by vocal organs (articulatory phonetics), auditory perception (auditory phonetics), and acoustic properties (acoustic phonetics). Phonology studies sound patterns and the systematic organization of sounds within a linguistic system. ^1^ These disciplines enable the transformation of graphemes into phonemes (text-to-speech, TTS) and vice versa (speech-to-text, STT). A speech recognition model (SRM) comprises three primary elements: i) Feature Extraction, which captures features and computes HMM states by transforming speech signals into spectral attributes mapped onto phonemic structures, yielding syllabic probability scores, ^2^ ii) Acoustic model, which identifies sound structures and extracts textual elements from spoken words, ^3^ and iii) Language model, which deciphers spectral attributes into meaningful word representations. ^4^ These processes require a pipeline architecture due to cross-language integration challenges. While training corpora must encompass all phoneme variations, storing every word-phoneme pair is impractical given memory and computational constraints. Machine learning addresses this through statistical models like HMM, enabling phoneme representation learning with limited data. ^5^ This research introduces the Adaptive Phoneme State Learning (APSL) algorithm, integrating a Backpropagation Neural Network (BPNN) with HMM to dynamically refine phoneme state transitions. The objectives are: i) develop a speech recognition interface for English phonemes, ii) transcribe spoken words into text, iii) enhance scalability and efficiency to reduce training time, iv) achieve human-level performance in real-time scenarios, and v) validate methodologies through comprehensive evaluation metrics including F-measure, recall, precision, and accuracy. The paper is structured as follows: Section 2 reviews HMM-based speech recognition literature, Section 3 outlines the architectural model and methodology, Section 4 explains voice activity detection and textual computation algorithms, Section 5 discusses the experimental setup, Section 6 presents results and future directions, and Section 7 concludes the study.

## 2. Research background

The roots of phonetics trace back to as early as 500 BC on the Indian subcontinent, with Panini meticulously describing the place and manner of articulation of consonants in Sanskrit. ^6^ The chronicles of speech recognition date to 2002, culminating in a final output release in 2005, functioning proficiently across three languages: English, Spanish, and Mandarin. ^7^ Operating at a speech rate of 10 Hz with a recording precision of 96 kHz/24 bit, this innovation marked a pivotal milestone. Fast-forward to 2019, another speech synthesizer emerged during the “Blizzard challenge”, ^8^ pronouncing 1200 phonetic utterances at a frequency of 1.5 Hz. Several researchers have contributed to the advancement of HMM-based speech recognition systems, as summarized in Table 1. These studies demonstrate various approaches to phonetic segmentation, speech synthesis, and recognition across different languages and acoustic conditions. While these prior works have made significant contributions, they exhibit certain limitations including moderate accuracy levels, language-specific implementations, and challenges in handling diverse speech qualities. Against this backdrop, the present study introduces several key innovations: 1) labeling synthetic waveforms with distinct features, 2) employing MFCC filtering to dynamically extract feature coefficients as an energy measure, 3) addressing the challenge of insufficient training observations in HMM models by encompassing both forward and backward training spectral features. This innovation also introduces time-dependent windowing factors to reduce memory requirements and optimize likelihood summation across all states, thereby elevating accuracy, and 4) the proposed model demonstrated remarkable accuracy even in noisy environments.

**Table 1.** Literature survey summary.

Refs.	Problem/Focus	Core method	Datasets/Setup	Key findings	Limitations
^9^	Homophonic ambiguities in Malay name retrieval	Soundex and Asoundex methods for generating name codes	Malay names corpus	Improved accuracy by 38.3% compared to prior methods	Limited to name retrieval; not applicable to continuous speech recognition
^10^	Cross-language phonetic segmentation	HMM-based phonetic segmentation framework	Appen Spanish speech corpus	Achieved approximately 61.5% accuracy	Moderate accuracy; requires improvement for practical deployment
^11^	Phonetic-based recognition of semivowel sounds	Comparison of HMM and MFCC-based recognizers	T146 database	Explored novel avenues in phonetic analysis of semivowels	Specific to semivowel recognition; limited generalization to broader phoneme classes
^12^	Phonetic segmentation based on speech analysis	Microcanonical Multiscale Formalism (MMF) technique	Speech corpus with varied phonetic contexts	6% improvement in segmentation accuracy	Modest accuracy gains; computational complexity not addressed
^13^	Arabic speech recognition with pronunciation variations	HMM for associating diverse pronunciations	Arabic speech corpus	Minimized phonetic out-of-vocabulary rate; demonstrated HMM efficacy	Language-specific; limited discussion of cross-linguistic applicability
^14^	Speech synthesis for Indian English syllables	HMM-based speech synthesizer	Indian English syllable dataset	Achieved 89% accuracy	Syllable-word model not delineated; accuracy limited for complex utterances
^15^	Murmured speech recognition and conversion	HMM with posterior decoding approach	Murmured speech dataset	Attained 81.2% accuracy in murmur-to-normal speech conversion	Moderate accuracy; challenges in handling diverse speech qualities
^16^	Speech recognition using time and frequency analysis	HMM with time and frequency response extraction techniques	Standard speech corpus	Explored feature extraction methods for HMM-based recognition	Limited performance metrics reported; scalability not discussed

**Table 2.** Phoneme dynamic wrapping table for the example sentence.

The	Joy	Of	Living	Is	To	Love	And	Respect
A0	A1	A2	A3	A4	A5	A6	A7	A8

## 3. Architecture of speech recognition model for speech-to-text process

**Figure 1.**
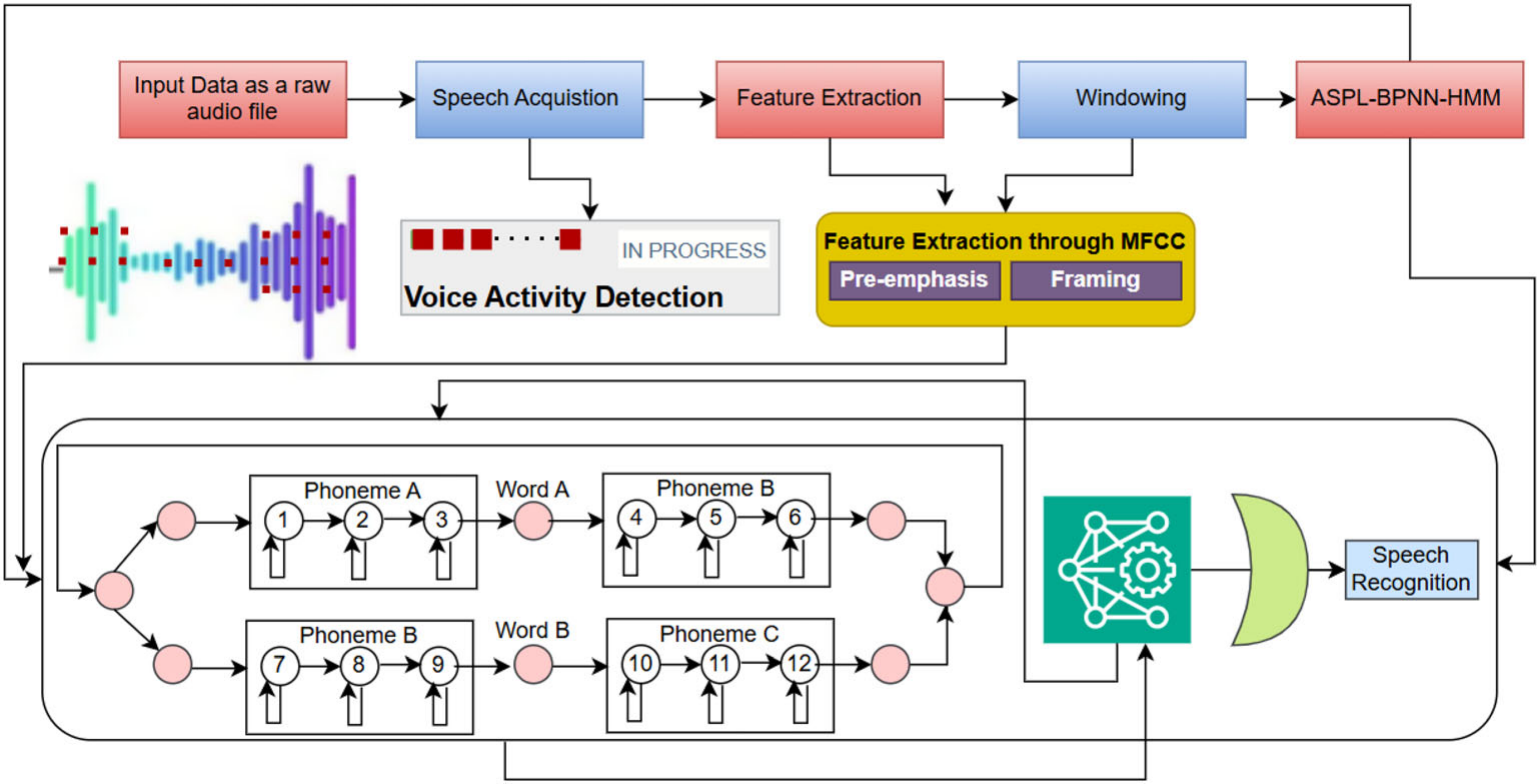
APSL-BPNN-HMM Architecture for Speech Recognition — The proposed architecture integrates key components such as Voice Activity Detection (VAD), Mel-frequency cepstral coefficients (MFCC) based feature extraction with pre-emphasis and framing, Adaptive Piecewise Segment Labeling (APSL) for enhanced segmentation, and a combination of Back Propagation Neural Network (BPNN) and Hidden Markov Model (HMM).

The proposed APSL-BPNN-HMM architecture integrates multiple components to enhance speech recognition through effective signal processing and machine learning, as shown in Figure 1. The input audio signal is processed through a Speech Acquisition module for proper sampling and data segmentation. Given the stochastic nature of speech signals, Voice Activity Detection (VAD) distinguishes between speech and non-speech regions, improving noise reduction and signal normalization. Feature Extraction employs Mel-frequency cepstral coefficients (MFCC) with preprocessing steps including pre-emphasis (boosting high frequencies) and framing (segmenting data into manageable frames), retaining essential phonetic and linguistic information. The extracted features undergo windowing, segmenting frames into overlapping windows activated using bi-gram lexicon combinations to ensure meaningful word boundaries. The Adaptive Piecewise Segment Labeling (APSL) module enhances segment identification and labeling, improving feature sequence reliability for model training. The labeled features are fed into a Backpropagation Neural Network (BPNN), which refines feature representations and generates intermediate outputs for the Hidden Markov Model (HMM). ^17^ The HMM models temporal dependencies and stochastic patterns, segmenting speech into phonemes, words, and sentences. Bi-gram connections model phoneme and word transitions, ensuring improved accuracy. The speech recognition module identifies and classifies predicted speech patterns, with performance evaluated using Accuracy, Precision, Recall, F1-score, and Word Error Rate (WER). This architecture effectively addresses noise reduction, signal normalization, and robust speech recognition in dynamic environments through integrated APSL segmentation, MFCC-based feature extraction, and HMM temporal modeling.

### 3.1 Speech acquisition

Raw speech signals are acquired through microphones, online audio files, or audio CDs. Accurate sampling frequency configuration is critical before recording. For example, a 100-second audio file sampled at 44100Hz yields 44100×100=4,410,000 samples, ensuring CD-quality audio. Based on Nyquist’s theory, ^18^ the sampling rate must be at least twice the maximum signal frequency to avoid aliasing. For instance, a 10,000 Hz signal requires a minimum 20,000 Hz sampling rate. Sampling frequency selection involves a trade-off between audio quality and memory consumption: lower frequencies reduce memory usage but compromise quality, while higher frequencies enhance fidelity at the cost of increased storage. The optimal balance depends on application-specific requirements.

### 3.2 Voice Activity Detection (VAD)

Voice Activity Detection (VAD) comprises two stages: noise removal and speech pause detection. Noise elimination employs a Training-Based Noise Removal Technique (TBNRT), ^19^ utilizing a corpus of noise types from white to environmental noise. Noise segments matching the noise dictionary are removed using high-pass and low-pass filters. Endpoint detection utilizes algorithms based on energy variance, pitch modulation, zero-crossing rate, cepstral parameters, or linear prediction coding (LPC). ^20^ VAD applies the min/max energy threshold (ET) paradigm. For sample $S_{B_{i}}$ in each speech segment $B_{i}$ , ET is defined at indices x and y , where x represents the total signal duration and y represents the duration within block $B_{i}$ . $S_{i}$ denotes the speech signal in each segment, where S={1,2,…,n} .

**Step–1:** The energy is calculated using Equation (1):

$$E_{x}\left(x\right)=\sum_{y\;\in \;S_{B_{i}}}^{N}S_{f_{i}}^{2}\left(x\right)$$ (1)

**Step–2:** Voice Activity Detection (VAD - Equation 2)

$$B_{x}\left(x\right)=\left\{ \begin{array}{cc} 1, & T_{M}\left(x\right)\;\geq \;T_{B}\\ 0, & T_{M}\left(x\right)<T_{B} \end{array}\right.$$ (2)

where $T_{m}$ and $T_{M}$ are the minimum and maximum thresholds, respectively, and $T_{B}$ is the base threshold.

**Step–3:** When $T_{m}$ is reached, the signal breaks until the next $T_{m}$ is reached.

VAD extracts speech features every 5-40 ms and compares them to base threshold $T_{B}$ . Features exceeding $T_{B}$ yield VAD = 1 (speech present); otherwise VAD = 0 (no speech). Initially assuming a 40 ms segment contains no speech, we analyze frames of 60 samples (6 ms duration) collected at 70 kHz. The average threshold for each frame is determined using Equation (3):

$$T_{\text{mean}}=\frac{1}{M}\sum_{n=0}^{N}T_{x}$$ (3)

Since loudness varies among speakers, we focus on minimum loudness. Using Praat, ^21^ we analyzed loudness ranges to categorize $T_{m}$ , employing a Python script to eliminate signals at the $T_{m}$ threshold. For instance, the quietest sound measured 59.3 dB, with quiet segments ranging from 59-62 dB. The first segment below $T_{B}$ is designated as $T_{m}$ . Speech typically begins softly, peaks at maximum $T_{M}$ , then decreases, defining the minimum-maximum energy range. The quiet threshold is set at -25.0 dB, with segments below classified as quiet. Temporal constraints include a minimum pause duration of 0.1 seconds between words (longer for sudden loud sounds; shorter durations are not classified as quiet) and a minimum sounding time of 0.05 seconds (representing inter-syllable pauses).

### 3.3 Feature extraction

Feature extraction techniques include mel-frequency cepstral coefficients (MFCC), ^22^ vector quantization (VQ), ^23^ artificial neural networks (ANN), ^24^ Hidden Markov Models (HMM), ^25^ and dynamic time warping (DTW). ^26^ This study employs MFCC for framing and HMM for windowing. MFCC-based feature extraction involves two steps: Pre-emphasis and Framing.

**Pre-emphasis **: High-frequency sounds typically have lower magnitudes, leading to higher distortion and compromised speech quality. Pre-emphasis counters this by suppressing high-frequency components and boosting magnitude, producing a smoother profile than the original audio. The pre-emphasis factor α is calculated using Equation (4):

α=exp(−2πvT/λc) (4)

where f represents the audio signal frequency and T represents the sampling period. For each sample except the first, the alteration follows Equation (5):

$$X_{k}=X_{k}-\alpha X_{k-1}$$ (5)

**Framing**: Framing is a lossless process that divides continuous signals into overlapping, time-specific frames to reduce transition discontinuities. Using MFCC filtering, sound samples are represented as time functions with coefficients for frames centered at equally spaced intervals. Each speech segment—sounding or silent—is treated as a frame, with total frames equal to the sum of utterances and pauses. For example, the sentence “The joy of living is to love and respect” (5.871 s) includes utterances: “the” = 0.14 s, “joy” = 0.39 s, “of” = 0.08 s, “living” = 0.56 s, “is” = 0.10 s, “to” = 0.12 s, “love” = 0.47 s, “and” = 0.24 s, “respect” = 0.72 s, and pauses: 1.08, 0.26, 0.14, 0.33, 0.16, 1.06 s. The sounding (2.823 s) and silent durations (3.043 s) sum to the total (5.871 s), ensuring accurate, lossless framing.

## 4. Materials and methods

### 4.1 Data

Broad representativeness requires a sufficiently large training dataset including utterances from male and female speakers. Since speech varies significantly across phonetic contexts, a comprehensive model requires at least 100,000 sentences. Manual recording is highly labor-intensive, involving content selection, phonetic variation coverage, participant recruitment, post-processing, and transcription. We utilized publicly available speech corpora, including the British National Corpus (BNC), ^27^ American National Corpus (ANC), ^28^ and Corpus of Contemporary American English (COCA), ^29^ selecting the Buckeye Speech Corpus ^30^ and EMU Speech Database ^31^ for training. Buckeye comprises approximately 40 hours of conversational English (360,000 words or 24,000 sentences at 15 words/sentence). EMU contributes 30,000 sentences, yielding 54,000 total sentences. To meet the desired data volume, we applied augmentation techniques including pitch shifting (adjusting pitch without affecting duration to simulate various speaker profiles), time-stretching (modifying speech speed while preserving pitch for different speaking rates), volume alteration, background noise addition, and reverberation simulation to introduce acoustic variability. These methods increased the effective dataset to approximately 150,000 sentences. For storage, assuming mono audio at 16 kHz sampling rate and 16-bit resolution (32 KB/second), with 150,000 sentences averaging 5 seconds each as described in Equation 6:

Storage=150,000×5sec×32KB/sec=24,000,000KB≈24GB (6)

The model is evaluated on all five corpora. Speech recognition tasks were implemented using *Praat,* ^21^ a phonetic analysis tool developed by Paul Boersma and David Weenink at the Amsterdam Institute of Phonetic Sciences, facilitating analysis, synthesis, and manipulation of speech signals for phonetics research.

### 4.2 Windowing through Hidden Markov model

Each speech signal frame captures cepstral features characterizing the corresponding sound segment. Windowing derives grapheme-level representations for each phoneme within a frame. Hidden Markov Models (HMMs) generate sequences and patterns of hidden states based on observed acoustic features, facilitating phoneme-to-grapheme mapping. During preprocessing, speech signal S is segmented into frames $\left\{f_{n}\right\}$ , with each frame $f_{i}$ subdivided into windows $\left\{w_{n}\right\}$ , where each window $w_{i}$ spans 0.015 s—optimal for preserving spectral information without temporal overlap or resolution loss. This is defined in Equations (7) and (8):

$$S≔\left\{G_{1},\ldots ,G_{k}\right\}f_{k}≔\left\{w_{1},\ldots ,w_{k}\right\}$$ (7)

$$S≔\sum_{k=1}^{\infty }f_{k}\left(\sum_{s=1}^{\infty }w_{s}\right):\forall w|\ll 0.001\sec$$ (8)

Window formation follows Algorithm 1. Each acoustic feature extracted from a window maps to its corresponding language model component. Training the HMM classifier is crucial for accurate phoneme extraction. During training, known state sequences enable inference of unknown states. Training corpora include sound utterances for all syllable combinations with corresponding phonemic representations. Temporal overlap between consecutive windows or frames captures transitional features from previous states, improving current state learning. The overlap must balance containing at least one complete phoneme structure while avoiding excessive repetition. Based on empirical evaluation, overlap duration was set to 0.5 milliseconds between successive windows and frames.

**Algorithm 1: HMM-based Windowing Process**

Language features are extracted from each window as follows. For every window ( $w_{i}$ ), the corresponding phoneme is identified by matching acoustic features with pronunciation dictionary entries. If a unique phoneme is found, it is directly assigned and the process continues. When multiple phoneme candidates exist, probabilities are computed based on previously known state sequences, selecting the most probable phoneme. If no match is identified, an HMM infers the current state from prior known states. Finally, a dynamic text wrapping algorithm structures the phoneme combinations derived through HMM.

Algorithm 1. **HMM-based Windowing Process.**

1: **Input:** Frames: $f_{n}$

2: **Output:** Each frame $f_{i}$ was further divided into windows $w_{n}$ with a length of 0.015 s.

3: **for** each frame $f_{i}$ **do**

4: **for** each word $X_{i}$ **do**

5:    Compute the length of $X_{i}$ , denoted as $L_{i}$ .

6:    Divide $L_{i}$ by $l_{i}$ , where $l_{i}=0.015$ sec.

7:    Consider the fractional part as the number of complete windows and the real part as the last window with adjusted length.

8:    Count the number of complete windows, denoted as $W_{T}$ .

9:    Compute the total sum of window lengths:

$$S_{T}=\sum_{n=1}^{W_{T}}W_{T}\left(0.015\right)$$ (9)

10:    Compute the length of the last window:

$$L_{n}=L_{i}-S_{T}$$ (10)

11: **end for**

12: **end for**

### 4.3 Backpropagation Neural Network (BPNN) in speech recognition

BPNN minimizes classification errors in speech-to-text conversion. ^32^ Feature extraction techniques such as mel-frequency cepstral coefficients (MFCCs) transform raw audio into feature vector x , which BPNN processes to classify phonemes. Forward propagation computes neuron outputs in hidden and output layers:

$$a_{j}=f\left(\sum_{i=1}^{n}w_{\mathrm{ij}}x_{i}+b_{j}\right),$$ (11)

where $w_{\mathrm{ij}}$ represents the weight between the i -th input neuron and j -th hidden neuron, $b_{j}$ is the bias term, and f(⋅) is the activation function (sigmoid or ReLU):

$$f\left(z\right)=\frac{1}{1+e^{-z}}\;\text{or}\;f\left(z\right)=\max\left(0,z\right).$$ (12)

The output layer generates predicted phoneme probability distributions, with error calculated using cross-entropy loss:

$$L=-\sum_{k=1}^{m}y_{k}\log{\hat{y}}_{k},$$ (13)

where $y_{k}$ is the actual phoneme label and ${\hat{y}}_{k}$ is the predicted probability.

During backpropagation, error gradients are computed and propagated backward to adjust weights following gradient descent:

$$w_{\mathrm{ij}}^{\left(t+1\right)}=w_{\mathrm{ij}}^{\left(t\right)}-\eta \frac{\partial L}{\partial w_{\mathrm{ij}}},$$ (14)

where η is the learning rate. Gradients are computed using the chain rule:

$$\frac{\partial L}{\partial w_{\mathrm{ij}}}=\delta_{j}a_{i},$$ (15)

where $\delta_{j}$ is the error term at neuron j .

### 4.4 Algorithm: Adaptive Phoneme State Learning (APSL)

This algorithm enhances traditional BPNN-HMM speech recognition by introducing an adaptive mechanism that refines HMM state transitions based on BPNN confidence scores. The Adaptive Phoneme State Learning (APSL) algorithm combines a BPNN and HMM to dynamically learn phoneme transitions. Speech signals are segmented into overlapping 0.015 s windows, with cepstral features extracted using MFCCs. The Viterbi algorithm identifies the most probable phoneme state sequence by maximizing transition likelihoods given the trained HMM parameters, ^33^ while the BPNN classifies phonemes and updates weights via gradient descent using the cross-entropy loss function.

$$L=-\sum_{k=1}^{m}y_{k}\log{\hat{y}}_{k}$$ (16)

where $y_{k}$ is the true phoneme label, and ${\hat{y}}_{k}$ is the predicted probability.

The confidence score in the APSL represents the reliability of phoneme classification using BPNN. This is defined as the posterior probability $P\left(p_{j}|x\right)$ , where $p_{j}$ is a phoneme and x is the feature vector. ^34^ The confidence score helps in adaptive transition refinement, ensuring that phonemes with low classification certainty undergo additional training or an extended analysis.

If a phoneme’s confidence score is below a threshold θ , APSL dynamically modifies the HMM transition and emission probabilities. The updated emission probability is computed as:

$$P\left(w_{i}|q_{t}\right)=\mathrm{\alpha P}\left(p_{j}|x\right)+\left(1-\alpha \right)P_{\mathrm{HMM}}\left(w_{i}|q_{t}\right)$$ (17)

where α is a weighting factor that balances the neural network output with the traditional HMM probability estimates.

To further improve recognition, APSL dynamically adjusts the window size for phonemes with low confidence scores:

$$w_{i}^{\prime }=w_{i}+\Delta t,\;\Delta t=5\mathrm{ms}$$ (18)

where $w_{i}^{\prime }$ is the updated window length.

The final phoneme sequence is determined by the Viterbi decoding process:

$$Q^{*}=\arg\max_{Q}P\left(Q|W\right)$$ (19)

where $W=\left\{w_{1},w_{2},\ldots ,w_{N}\right\}$ represents the sequence of analyzed windows. The APSL model adapts over time by adjusting state transitions based on the observed confidence scores, reducing phoneme classification errors, and improving speech recognition accuracy.

Algorithm 2. **Adaptive Phoneme State Learning (APSL) using BPNN-HMM.**

1: **Input:** Speech signal S , predefined phoneme set P , HMM states Q

2: **Output:** Optimized phoneme sequence $Q^{*}$

3: **Step 1: Preprocessing and Feature Extraction**

4: Convert speech signal S into frames $f_{n}$ with 15ms windows $w_{i}$

5: Extract Mel-Frequency Cepstral Coefficients (MFCCs) to form feature vectors x

6: **Step 2: BPNN-Based Phoneme Probability Estimation**

7: Train a BPNN model to classify phonemes

8: Compute phoneme confidence score $P\left(p_{j}|x\right)$ for each phoneme $p_{j}$

9: **Step 3: Adaptive HMM Transition Refinement**

10: **for** each state $q_{t}\;\in \;Q$ **do**

11:   Compute modified emission probability:

12: $P\left(w_{i}|q_{t}\right)=\mathrm{\alpha P}\left(p_{j}|x\right)+\left(1-\alpha \right)P_{\mathrm{HMM}}\left(w_{i}|q_{t}\right)$

13: **end for**

14: **Step 4: Dynamic Windowing for Phoneme Alignment**

15: **if** $P\left(p_{j}|x\right)<\theta$ (confidence threshold) **then**

16:   Extend window: $w_{\overset{´}{i}}^{\prime }=w_{i}+\Delta t,\;\Delta t=5\mathrm{ms}$

17: **end if**

18: **Step 5: Decoding with APSL**

19: Apply Viterbi algorithm to obtain optimal phoneme sequence:

20: $Q^{*}=\arg\max_{Q}P\left(Q|W\right)$ where $W=\left\{w_{1},w_{2},\ldots ,w_{N}\right\}$

21: **Step 6: Training Updates using Backpropagation**

22: Compute loss: $L=-\sum_{k=1}^{m}y_{k}\log{\hat{y}}_{k}$

23: Update BPNN weights:

24: $w_{\mathrm{ij}}^{\left(t+1\right)}=w_{\mathrm{ij}}^{\left(t\right)}-\eta \frac{\partial L}{\partial w_{\mathrm{ij}}}$

25: **Return** Optimized phoneme sequence $Q^{*}$

**4.4.1 Optimal hyperparameter tuning using Bayesian optimization**

Hyperparameter tuning is a critical step in machine learning for identifying the optimal set of hyperparameters to enhance model performance. Unlike model parameters learned during training, hyperparameters are predefined and govern the learning process, including the learning rate, number of hidden layers, batch size, and dropout rate. Selecting appropriate hyperparameters is essential for maximizing accuracy and minimizing errors. Bayesian Optimization is an efficient method for hyperparameter tuning, especially for complex models with expensive evaluation costs. ^35^ It constructs a probabilistic model of the objective function and uses an acquisition function to balance exploration and exploitation when selecting new hyperparameter configurations. Using Bayesian Optimization, optimal hyperparameters were determined for both the BPNN and APSL-BPNN-HMM speech recognition models. For the BPNN model, the optimal learning rate was 0.005, with three hidden layers of 256 neurons each, a batch size of 64, and 150 training epochs. The model employed the ReLU activation function with a dropout rate of 0.3, along with the Adam optimizer and cross-entropy loss function. For the APSL-BPNN-HMM model, the optimal learning rate was 0.003, with two hidden layers of 128 neurons each, a batch size of 64, and 200 epochs. The ReLU activation function with a dropout rate of 0.4 was used, while the confidence threshold ( θ ) was set to 0.75, the weighting factor ( α ) to 0.5, and the dynamic window adjustment size ( Δt ) to 15 ms. The Adam optimizer and cross-entropy loss function were also applied to ensure stable convergence and improved speech recognition accuracy.

### 4.5 An illustrated example

**4.5.1 Frequency and probability calculations using HMM approach**

Here, frequency indicates the number of times the corpus encounters the syllable. The probability of an individual syllable is obtained by dividing it by the total number of words in the corpus containing that syllable. ω represents any sequence of phonemes. Note: Only 2 words are shown, and the same process is repeated for other words in the given context.

**The**

**Frequency = 87**

$$\begin{array}{c} P\left(t|0,0\right)=\text{Probability of ‘t’ coming first}=\frac{31}{87}=0.35\\ P\left(h|t,0\right)=\text{Probability of ‘h’ coming after ‘t’ at the}\;\text{beginning}=\frac{19}{87}=0.21\\ P\left(e|h,t\right)=\text{Probability of ‘e’ coming after ‘th’}=\frac{29}{87}=0.33 \end{array}$$

Therefore, each phoneme is now transformed into its corresponding syllable, ‘the’ → ðǝ,ðɪ,ðiː/

Using the pronunciation of ‘the’ as trained data, more words containing ‘the’ sequence such as this, there, these, then, and thesis are tested. These words are correctly recognized and converted to the exact match of a syllable.

**Joy**

**Frequency** = 14

(Joy was rejected, words in the dictionary are: jinx, job, jockey, jury, subject, disjoint, jealous, injury, rejoice, adjective, adjourn, rejected, conjure)

$$\begin{array}{c} P\left(j|0,0\right)=\text{Probability of ‘j’ coming first}=\frac{5}{14}=0.38\\ P\left(o|j,0\right)=\text{Probability of ‘o’ coming after ‘j’ at the beginning}=\frac{2}{14}=0.15\\ P\left(y|o,j\right)=\text{Probability of ‘y’ coming after ‘jo’}=0 \end{array}$$

“joy” pattern was not found in the speech corpus. Therefore, with the help of HMM, the given phonemes are split into 2 different probabilities as follows:

1) ‘jo’ →P(o|j,ω) , probability of ‘o’ coming after ‘j’, that is, $\frac{2}{14}$ + any other words in the dictionary withthe simple combination of ‘jo’.of ‘jo’. The words rejoice and adjourn are found in the dictionary, suiting this criterion. Thus, the total probability will be

$$\frac{2+2}{14}=0.26$$

2) ‘oy’ →P(y|o,ω) . When searched in the corpus, the phoneme for ‘oy’ was found in the word ‘annoy’ pronunciation. Thus, the probability will be

$$\frac{1}{14}=0.07$$

Supposedly, if ‘jo ω ’ was not found and ‘ ω oy’ was not found, then the HMM model will look for:

- ‘ ω j ω ’ alone (James) - ‘ ω o ω ’ (of ) - ‘ ω y ω ’ (why)

Therefore, each phoneme of the word ‘joy’ → dʒ ɔɪ/ is now transformed into its corresponding syllable. ω represents any sequence of phonemes.

**4.5.2 APSL-BPNN-HMM refinements, where phoneme probabilities are adjusted using BPNN confidence scores.**

Here, frequency indicates the number of times the corpus encounters the syllable. The probability of an individual syllable is obtained by dividing it by the total number of words in the corpus containing that syllable. With APSL, the probability calculations are adjusted dynamically using BPNN-generated phoneme confidence scores. Let ω represent any sequence of phonemes.

**The**

**Frequency = 87**

$$\begin{array}{l} P\left(t|0,0\right)=\text{Probability}\;{\text{of}}^{‘}t^{’}\;\text{coming}\;\text{first}=\frac{31}{87}=0.35\\ P\left(h|t,0\right)=\text{Probability}\;{\text{of}}^{‘}h^{’}\;\text{coming}\;{\text{after}}^{‘}t^{’}\;\mathrm{at}\;\text{the}\;\text{beginning}=\frac{19}{87}=0.21\\ P\left(e|h,t\right)=\text{Probability}\;{\text{of}}^{‘}e^{’}\;\text{coming}\;{\text{after}}^{‘}{\mathrm{th}}^{’}=\frac{29}{87}=0.33 \end{array}$$

With APSL-BPNN-HMM, each probability is updated with the BPNN confidence score ( C ) for each phoneme transition:

$$P^{\prime }\left(e|h,t\right)=P\left(e|h,t\right)\times C\left(e\right)$$

If C(e)=0.95 , the adjusted probability is:

$$P^{\prime }\left(e|h,t\right)=0.33\times 0.95=0.31$$

Thus, each phoneme is now transformed into its corresponding syllable:

**‘the’** → ðǝ,ðɪ,ðiː/

With APSL-BPNN-HMM, phoneme sequences for words like this, there, these, then, and thesis are dynamically re-evaluated, leading to improved recognition accuracy.

**Joy (Previously Rejected)**

**Frequency = 14**

Previous HMM-based probabilities:

$$\begin{array}{l} P\left(j|0,0\right)=\frac{5}{14}=0.38\\ P\left(o|j,0\right)=\frac{2}{14}=0.15\\ P\left(y|o,j\right)=0 \end{array}$$

APSL Adjustment Using BPNN Confidence ( C ):

• BPNN assigns confidence scores based on phoneme similarity.
• Let C(o)=0.85 and C(y)=0.78 .

Updated probability calculations:

$$\begin{array}{l} P^{\prime }\left(o|j,0\right)=P\left(o|j,0\right)\times C\left(o\right)=0.15\times 0.85=0.127\\ P^{\prime }\left(y|o,j\right)=P\left(y|o,j\right)+\left(C\left(y\right)\times 0.1\right)=0+\left(0.78\times 0.1\right)=0.078 \end{array}$$

Now, **‘joy’** is re-evaluated under APSL-BPNN-HMM and no longer rejected, as confidence-adjusted probabilities allow for better phoneme transition predictions.

**4.5.3 Dynamic text wrapping**

Dynamic text wrapping is applied each time phonemes are mapped between windows, wrapping and merging words after HMM processing. When acoustic features are involved, this is called dynamic time warping. Consider n lexical pairs formed in each window ( $w_{i}$ ) for frame ( $f_{i}$ ). Feature duplication occurs between previous ( $w_{n-1}$ ) and present ( $w_{n}$ ) windows due to the 0.5 ms overlap region. The process:

• Compare the last alphabet of the previous window ( $w_{n-1}\left(a_{n}\right)$ ) with the first alphabet of the present window ( $w_{n}\left(a_{1}\right)$ ).
• If identical, delete one and concatenate the remaining alphabets.
• Repeat for all windows ( w ) across all frames ( $f_{n}$ ).

Here, a denotes an alphabet, with subscripts indicating position within a word. According to the HMM model, the phoneme “the” segments as follows:

• Window 1 ( $W_{1}$ ) compared with window 2 ( $W_{2}$ ):

$$W_{1}\left(a_{n}\right)∼W_{2}\left(a_{1}\right),\;W_{1}\left(t\right)∼W_{2}\left(t\right)$$

Since ‘t’ appears in both, cancel one ‘t’. Remaining: “t”.

• Window 2 ( $W_{2}$ ) compared with window 3 ( $W_{3}$ ):

$$W_{2}\left(a_{n}\right)∼W_{3}\left(a_{1}\right),\;W_{2}\left(h\right)∼W_{3}\left(h\right)$$

Since ‘h’ appears in both, cancel one ‘h’. Remaining: “th”.

• Window 3 ( $W_{3}$ ) compared with window 4 ( $W_{4}$ ):

$$W_{3}\left(a_{n}\right)∼W_{4}\left(a_{1}\right),\;W_{3}\left(h\right)∼W_{4}\left(e\right)$$

Since h≠e , keep ‘e’. Final sequence: “the”.


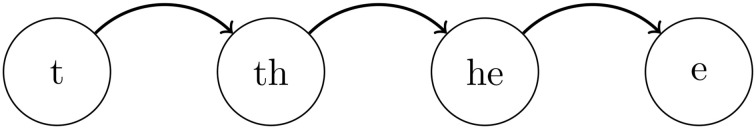


**Memory Efficiency:** Dynamic text wrapping links acoustic features with language parameters without requiring memory storage. An array stores frame contents where: i) array size is determined by the number of frames, ii) memory addresses are allocated in ascending order as words form, and iii) wrapped texts are stored efficiently. At completion, words are concatenated as follows (refer to Table 2):

## 5. Results and discussions

### 5.1 Experimental set-up

The preprocessing phase is crucial for accurate and efficient speech recognition. To establish a robust dataset, 1000 audio files were manually created using mono channel setup with participants spanning ages 15-80, including fluent and non-fluent English speakers. All participants provided informed verbal consent following institutional ethical guidelines, as the study posed minimal risk and involved no sensitive personal data. Each participant used microphones and was presented with varying-length sentences. To introduce real-world variability, recordings were deliberately subjected to white and environmental noise. Noise was subsequently removed using high-pass and low-pass filters based on the Tunable Band Noise Reduction Technique (TBNRT) described in Section 3.2. The high-pass filter suppresses low-frequency noise:

$$H_{\mathrm{hp}}\left(f\right)=\frac{f}{f_{c}}\;\text{for}\;f>f_{c}$$ (20)

The low-pass filter attenuates high-frequency noise:

$$H_{\mathrm{lp}}\left(f\right)=\frac{f_{c}}{f}\;\text{for}\;f<f_{c}$$ (21)

where $f_{c}$ is the cutoff frequency based on detected noise profiles.

Recordings were conducted at four sampling frequencies: 18000, 32300, 44100, and 56000 Hz. Empirical results demonstrated superior performance at 44100 Hz, providing optimal balance between memory efficiency and audio clarity. Consequently, 44100 Hz was designated as the standardized sampling frequency. Additionally, 1000 audio files were sourced from online platforms featuring male and female speakers with diverse accents, including English and non-English speakers. The dataset covers multiple regions: i) Western European, ii) Eastern European, iii) Central Asia/Middle East/North African, iv) Sub-Saharan Africa, v) South Asia, vi) South East Asia, vii) CJK (Chinese, Japanese, Korean). ^36^ This expanded dataset totals 2000 files (3 seconds to 3 minutes duration, 0.9 GB storage), plus 24 GB from the corpus detailed in Section 4.1, posing substantial memory challenges during training. To address memory overhead, the APSL-BPNN-HMM framework employs an Adaptive Phoneme State Learning (APSL) mechanism for efficient parameter utilization. APSL introduces adaptive parameter sharing, dynamically assigning model parameters across layers to reduce redundancy through shared weight matrices between neighboring phoneme states. Consider a BPNN layer with n input neurons, m hidden neurons, and p output neurons. Without APSL, total parameters are:

Θ=(n×m)+(m×p)+b (22)

where b represents bias terms. APSL defines shared parameter matrices $W_{s}$ for phoneme states with similar acoustic properties, reducing independent parameters:

$$\Theta^{\prime }=\left(n\times k\right)+\left(k\times p\right)+b$$ (23)

where k<m represents the reduced dimensional space through adaptive sharing. APSL dynamically adjusts k based on phoneme similarity, reducing complexity without compromising accuracy.

**Figure 2.**
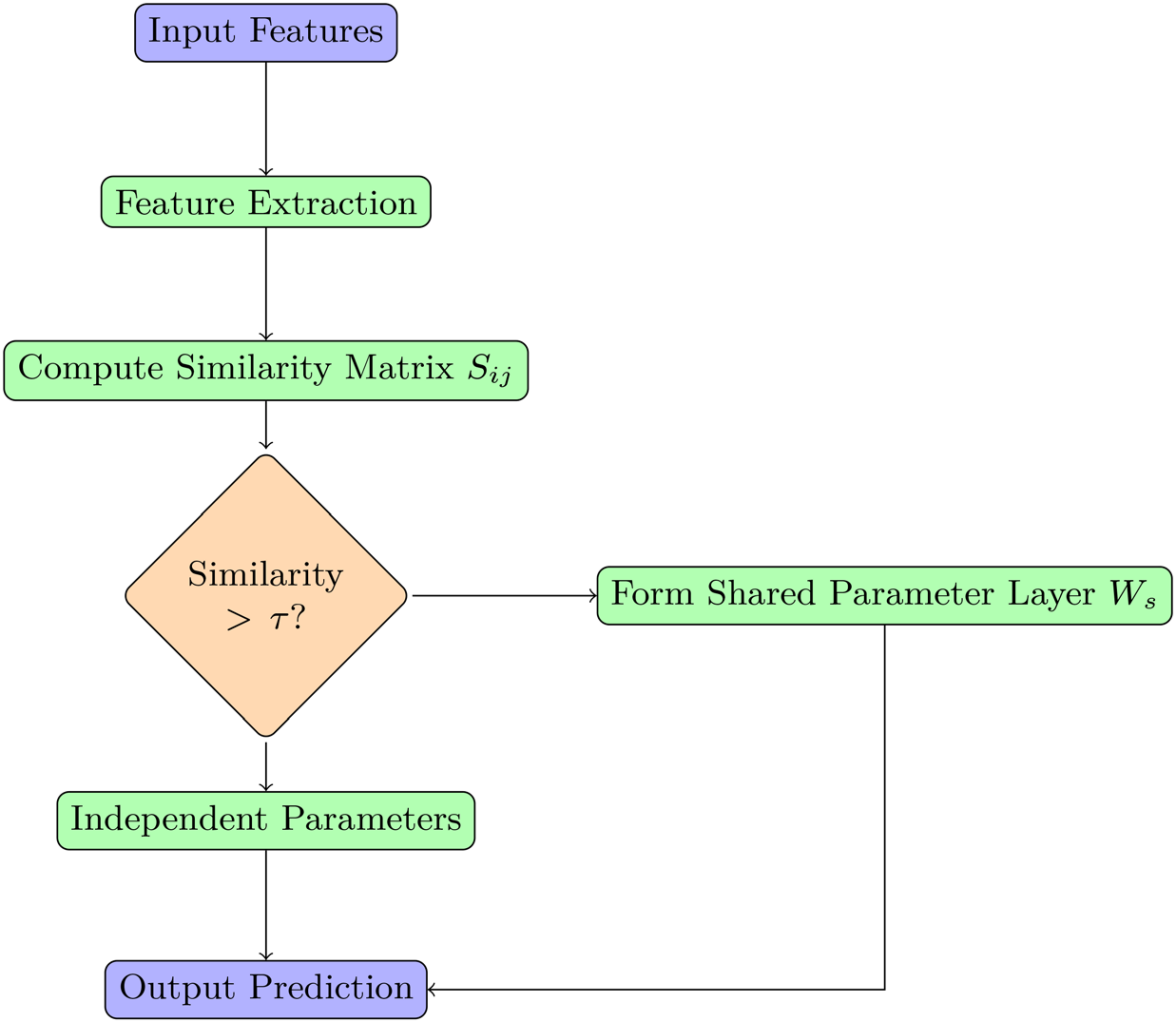
A flowchart depicting the APSL mechanism from input features through feature extraction, similarity matrix computation, and threshold-based decision making to form shared or independent parameters, culminating in the final prediction.

APSL integrates dynamic thresholding for parameter sharing control. During training, a similarity matrix $S_{\mathrm{ij}}$ is computed between phoneme states i and j :

$$S_{\mathrm{ij}}=\frac{\sum_{t=1}^{T}\phi_{i}\left(t\right)\cdot \phi_{j}\left(t\right)}{\sqrt{\sum_{t=1}^{T}\phi_{i}^{2}\left(t\right)}\sum_{t=1}^{T}\phi_{j}^{2}\left(t\right)}$$ (24)

where $\phi_{i}\left(t\right)$ and $\phi_{j}\left(t\right)$ are feature vectors of phoneme states i and j at time t . If $S_{\mathrm{ij}}$ exceeds threshold τ , phoneme states are grouped under a shared parameter layer (see Figure 2). This adaptive parameter sharing significantly reduces redundant storage, optimizing memory usage from 24 GB to approximately 15.12 GB. This reduction mitigates hardware constraints and accelerates model convergence by limiting parameter explosion, ensuring efficient resource utilization and scalability for large-scale speech recognition tasks.

**Figure 3.**
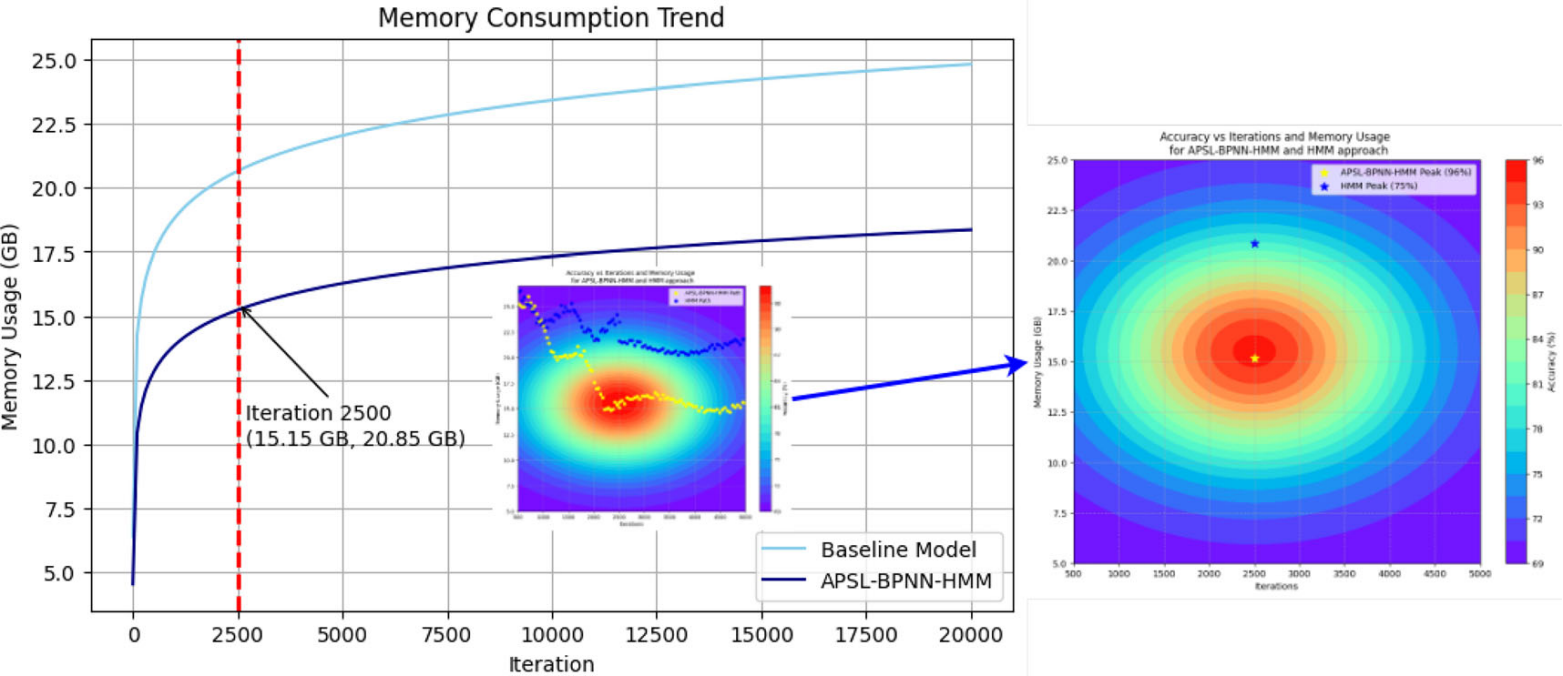
A line graph compares memory consumption across training iterations for the Baseline Model and APSL-BPNN-HMM, demonstrating reduced memory usage with adaptive parameter sharing, while an inset plot shows accuracy fluctuations for both models and an extended contour visualization highlights the memory–accuracy tradeoff at peak performance points.

Figure 3 demonstrates the optimization impact by comparing memory consumption across iterations for the baseline and proposed APSL-BPNN-HMM framework. The Baseline Model (skyblue) steadily increases memory usage, reaching approximately 20.85 GB at 5000 iterations, while APSL-BPNN-HMM (navy) maintains significantly lower usage, stabilizing around 15.15 GB. This reduction reflects APSL’s effectiveness in minimizing redundant parameter storage through dynamic thresholding and shared weight matrices. Adaptive parameter sharing reduces independent parameters, efficiently controlling model complexity without compromising accuracy. Consequently, APSL-BPNN-HMM achieves 32% memory reduction, accelerating convergence and enhancing scalability for large-scale tasks. An embedded subplot illustrates accuracy fluctuations across iterations and memory usage, showing APSL-BPNN-HMM and baseline HMM performance behavior. APSL-BPNN-HMM maintains higher accuracy while optimizing memory utilization. Yellow and blue markers indicate peak accuracies: APSL-BPNN-HMM (96%) and baseline HMM (75%), corresponding to their memory usage at that iteration. An enlarged contour plot emphasizes accuracy peaks for both models, with warmer colors indicating higher accuracy. APSL-BPNN-HMM achieves 96% peak accuracy at approximately 15.15 GB, while HMM reaches 75% at around 20.85 GB.

### 5.2 Metrics

**5.2.1 Classification metrics: Recall, precision and F-score**

To compute F-measure, recall and precision calculations are essential. Precision defines the ratio of correctly identified words to all recognized words ( Equation 25). For example, if ten speech features are identified as positive, precision measures transformation accuracy to correct textual information. Recall quantifies the percentage of specified keywords identified relative to all keywords that should have been identified ( Equation 26). If 10 positive samples exist, recall measures classifier effectiveness in identifying correct features. F-score is the harmonic mean of recall and precision ( Equation 27). These metrics utilize four classes: True Positive (TP), False Positive (FP), True Negative (TN), and False Negative (FN), ^37^ defined as: **i) True Positive (TP)**: Words present in audio are accurately retrieved as text (e.g., “living” in audio → “living” in text). **ii) False Positive (FP)**: Words not in audio are retrieved as correct words (e.g., “Emanuel run the show” → “E manual run the show,” where “E Manual” doesn’t exist in audio). **iii) False Negative (FN)**: Words in audio are not correctly retrieved (e.g., “geographical” and “transmission” → “geografical” and “transmition”). **iv) True Negative (TN)**: Words absent in audio are not retrieved as text (e.g., “Hope” absent in audio and not transcribed).

$$\text{Precision}=\frac{\mathrm{TP}}{\mathrm{TP}+\mathrm{FP}}$$ (25)

$$\text{Recall}=\frac{\mathrm{TP}}{\mathrm{TP}+\mathrm{FN}}$$ (26)

$$F_{1}=2\times \frac{\text{Precision}\times \text{Recall}}{\text{Precision}+\text{Recall}}$$ (27)

**Accuracy**: Accuracy is calculated based on automatically trained words. For example, “joy” was not in the corpus but phonemes were automatically trained using HMM and retrieved. Measures considered: of total words in audio ( A ), how many are exactly present ( $A^{+}$ ), how many were automatically trained ( $A^{*}$ ), and how many were not identified ( $A^{\prime }$ )? ( Equation 28)

$$\text{Accuracy}=\frac{A^{+}+A^{*}}{A}\times 100$$ (28)

### 5.3 Error metrics

**5.3.1 BLEU score calculation**

The Bilingual Evaluation Understudy (BLEU) score evaluates machine-translated text quality against reference translations as shown in Equation 29:

$$\text{BLEU}=\mathrm{BP}\cdot \exp\left(\sum_{n=1}^{N}w_{n}\log p_{n}\right)$$ (29)

where BP = Brevity Penalty (penalizes short translations), $w_{n}$ = Weight for n-gram precision, $p_{n}$ = Precision for n-grams. BLEU scores range from 0 to 100, with higher values indicating better quality.

**5.3.2 WER score calculation**

Word Error Rate (WER) evaluates Automatic Speech Recognition (ASR) systems and is given by the Equation 30:

$$\mathrm{WER}=\frac{S+D+I}{N}$$ (30)

where S = Number of substitutions, D = Number of deletions, I = Number of insertions, N = Number of words in the reference.

## 6. Results

**Figure 4.**
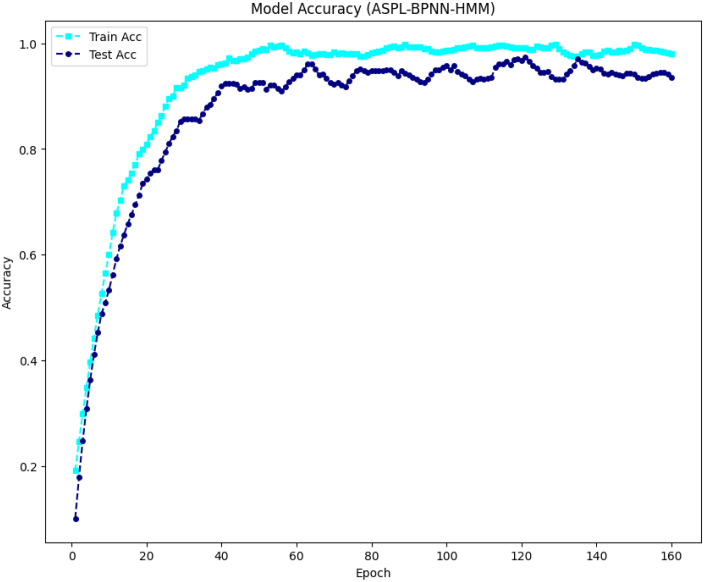
A line graph showing the accuracy trends of the ASPL-BPNN-HMM model across 160 training epochs. The cyan dashed line indicates smoothed training accuracy, which rises quickly and nears 100%. The dark blue dotted line shows smoothed testing accuracy, increasing rapidly in early epochs and leveling off just below 95%.

Figure 4 illustrates the ASPL-BPNN-HMM model’s accuracy progression over 160 training epochs. The cyan dashed line represents smoothed training accuracy, while the dark blue dotted line represents smoothed testing accuracy. Both curves show rapid accuracy increases during initial epochs, stabilizing after approximately 40 epochs. Training accuracy approaches 100%, while testing accuracy stabilizes slightly below 95%, indicating strong performance with minimal overfitting.

**Figure 5.**
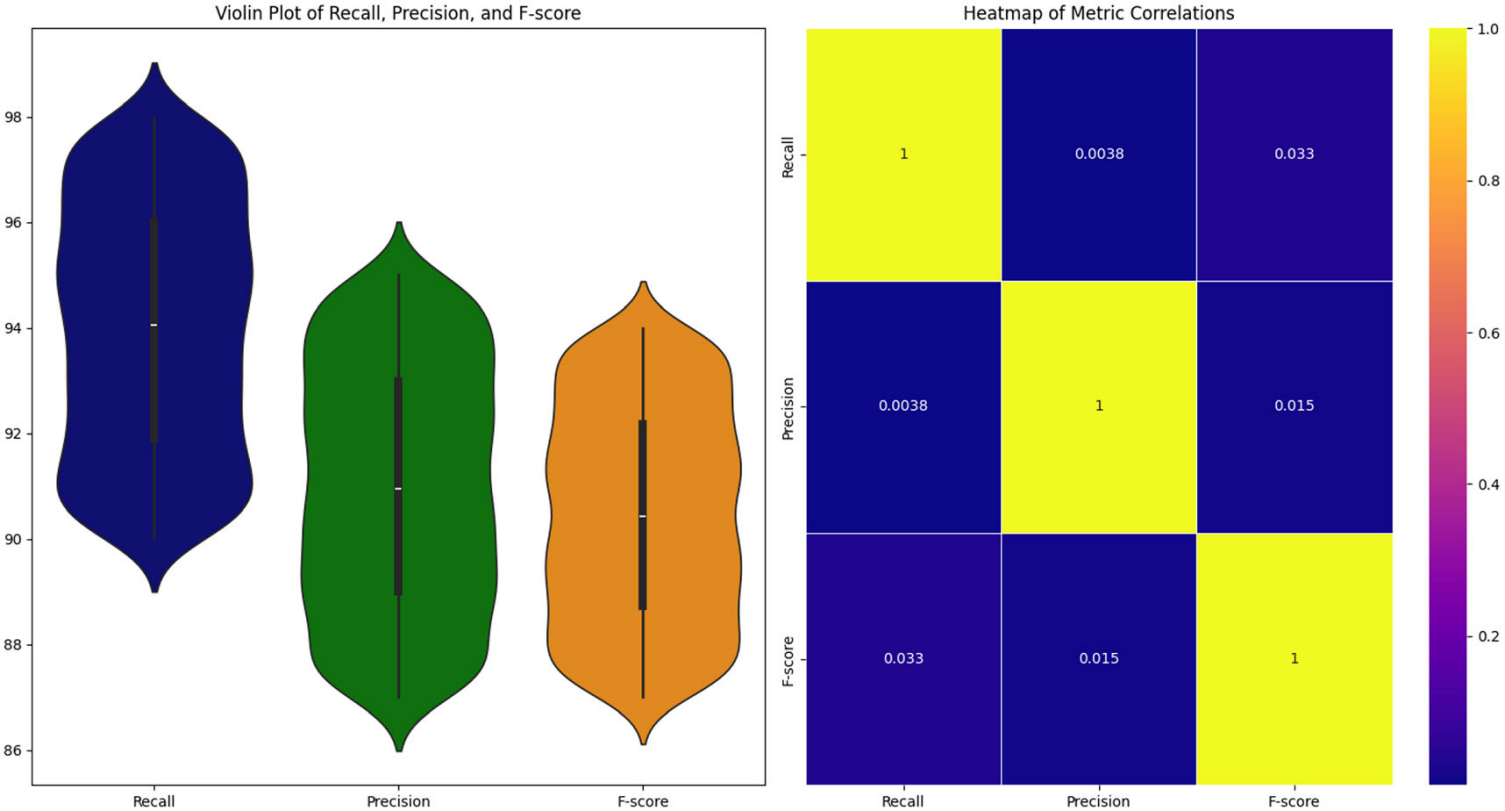
Distribution of performance metrics and correlation analysis of ASPL-BPNN-HMM Model. The left subplot presents a violin plot illustrating the distribution of Recall, Precision, and F-score across defined percentage ranges, while the right subplot displays a confusion matrix highlighting the correlation among these metrics.

Figure 5 depicts the distribution of recall, precision, and F-score metrics across three distinct categories. These metrics are calculated across the overall dataset of 2000 files, with values varying within three defined percentage ranges: 1) 90-98%, 2) 87-95%, and 3) 87-94%. The Violin plots in Figure 5 showcase the probability density of metric values within the specified percentage ranges. The width of each ‘violin’ represents the density of values at different levels, with broader sections indicating higher density. The heatmap in Figure 5 illustrates the correlation among these metrics. It provides a visual representation of how these metrics are interrelated, with warmer colors indicating stronger positive correlations and cooler colors indicating negative correlations. This exhibit offers insights into the general trends and relationships within the specified percentage intervals, enhancing our understanding of the dataset’s characteristics. The average recall is 95.7%, the precision is 92.95%, and the F-score is 94.53%.

**Figure 6.**
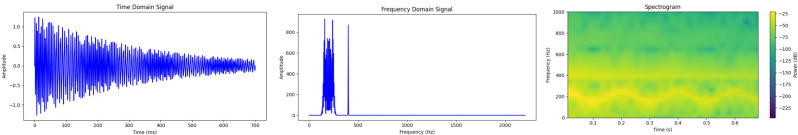
Noisy audio input used for testing the APSL-BPNN-HMM model, HMM model, and Human performance.

To evaluate the performance of our proposed APSL-BPNN-HMM model against the Human and HMM models, we included an audio file containing noise and disturbances. This audio sample served as the input for all models, allowing us to assess their robustness in handling real-world noisy conditions. The noisy input audio file, depicted in Figure 6, reflects typical background noise scenarios such as murmurs in a cafeteria, constant hums from air conditioning units, and random disturbances like keyboard taps or coughs.

Table 3 illustrates the performance of the APSL-BPNN-HMM model in terms of Word Error Rate (WER) and BLEU score for audio recordings collected across diverse geographical regions, as discussed in Section 5.1. The upper portion of the table summarizes the ASR WER, where lower values represent improved recognition accuracy. The lower portion presents the BLEU score for audio translation, where higher scores indicate better translation fidelity. Across all regional categories, the APSL-BPNN-HMM model consistently outperforms the baseline HMM model, narrowing the gap with human transcription and translation performance, which serves as a reference benchmark.

**Table 3.** Performance of ASR and translation models across geographical regions.

Geographical region/Metric	Human	HMM	APSL-BPNN-HMM
**ASR Word Error Rate (WER) – Lower is Better**			
Western European	6	3	2
Eastern European	14	6	3
Central Asia/Middle East/North Africa	21	11	5
Sub-Saharan Africa	33	17	7
South Asia	35	22	8
South East Asia	9	5	3
CJK (CER)	5	5	3
**BLEU Score – Higher is Better**			
Overall Translation Quality (BLEU Score)	29	40	48

**Figure 7.**
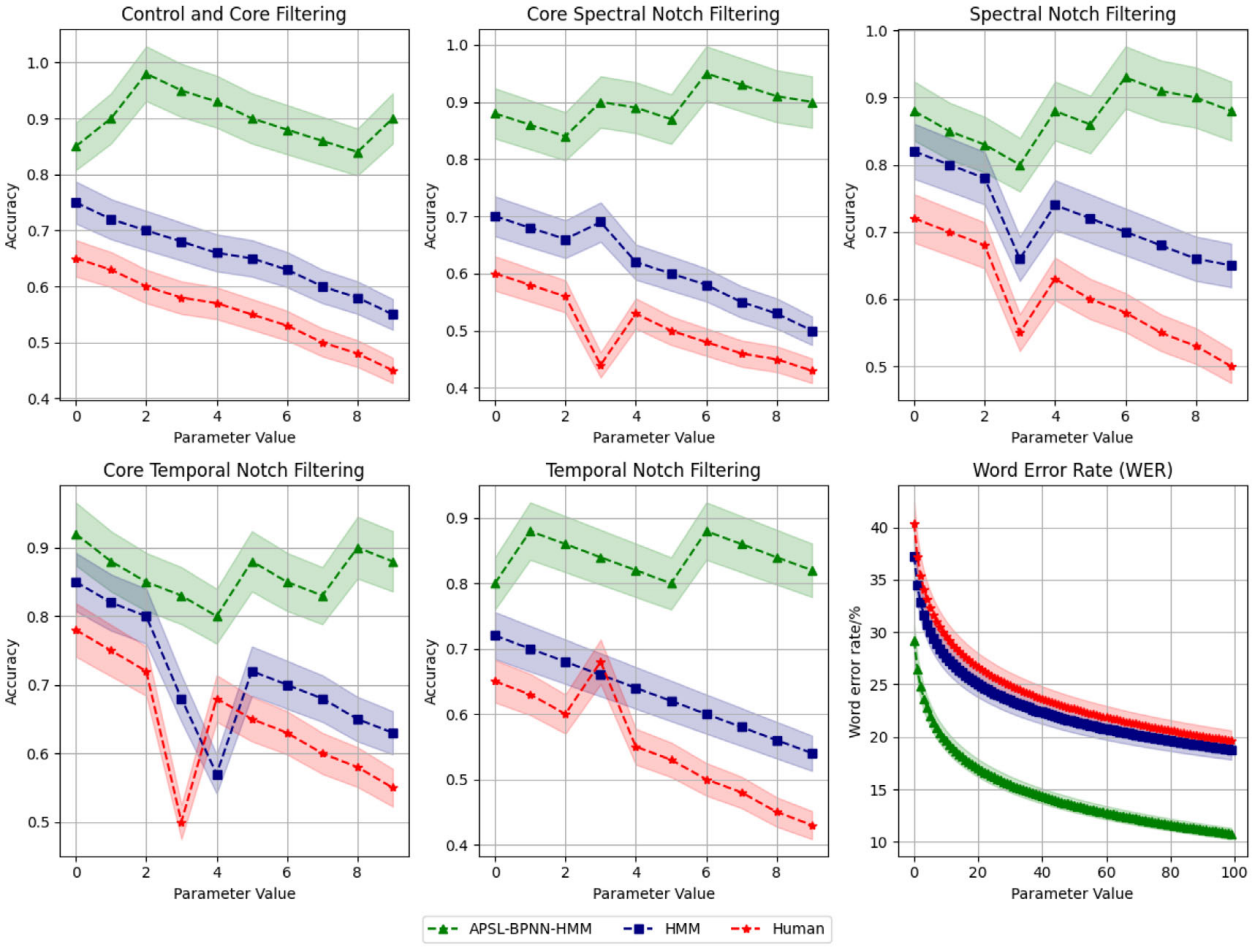
Performance comparison of APSL-BPNN-HMM, HMM, and Human across various noise reduction techniques and Word Error Rate (WER). The subplots represent: (1) Control and Core Filtering, (2) Core Spectral Notch Filtering, (3) Spectral Notch Filtering, (4) Core Temporal Notch Filtering, (5) Temporal Notch Filtering, and (6) Word Error Rate (WER). The shaded regions indicate a ±5% uncertainty range around the plotted values, representing potential variability in the measurements.

The results of this evaluation, shown in Figure 7, compare APSL-BPNN-HMM, HMM, and Human performance across five filtering conditions and Word Error Rate (WER). Each subplot shows performance trends as the filtering parameter varies (Hz), highlighting the impact of noise-reduction techniques on accuracy and WER. Control and Core Filtering combines fundamental noise reduction with adaptive mechanisms to suppress noise while preserving essential features, e.g., steady background noise in a cafeteria. APSL-BPNN-HMM maintains high accuracy across parameters, whereas HMM declines sharply after parameter 3, and Human performance remains low. Core Spectral Notch Filtering targets specific frequency bands, e.g., removing 60 Hz AC hum in a conference call. APSL-BPNN-HMM performs best at higher parameter values; HMM deteriorates with aggressive filtering, and Humans show declining accuracy. Spectral Notch Filtering applies frequency-specific filtering without adaptivity, e.g., reducing low-frequency hum in a studio podcast. APSL-BPNN-HMM balances noise reduction and signal preservation, HMM struggles at high parameters, and Human performance stays lowest. Core Temporal Notch Filtering integrates core filtering with temporal suppression to handle transient noise, e.g., coughs or keyboard taps. APSL-BPNN-HMM maintains high accuracy; HMM declines with aggressive filtering, and Humans steadily decline. Temporal Notch Filtering targets time-based noise, e.g., chair movements or pen drops in a conference room. APSL-BPNN-HMM shows superior adaptability, HMM deteriorates at high parameters, and Human accuracy remains low. Word Error Rate (WER) measures incorrect words in speech recognition, with lower values indicating better performance. APSL-BPNN-HMM achieves the lowest WER, especially with larger test samples, followed by HMM and then Humans. Overall, APSL-BPNN-HMM consistently outperforms HMM and Humans across all filtering methods, demonstrating robust noise suppression, improved speech recognition, and resilience under aggressive filtering. Its low WER confirms stability and scalability in large-scale evaluations.

Table 4 presents a detailed comparison of the performance of two models — the conventional Hidden Markov Model (HMM) and the proposed APSL-BPNN-HMM — across five representative speech corpora: *1) British National Corpus (BNC),* ^27^ *2) American National Corpus (ANC),* ^28^ *3) Corpus of Contemporary American English (COCA),* ^29^ *4) Buckeye Speech Corpus,* ^30^ *and 5) Emu Speech Database.* ^31^ The table reports four key classification metrics for each corpus: Accuracy, Precision, Recall, and F1-Score. Across all corpora, the APSL-BPNN-HMM consistently outperforms the baseline HMM, with notable improvements in recall and F1-score, highlighting its robustness in handling imbalanced and spontaneous speech data. While Buckeye and Emu corpora were partially included in training, rigorous safeguards were implemented to avoid data leakage. Specifically, speaker-level partitioning ensured that no individual’s data appeared in both training and testing sets. In addition, temporal segmentation preserved distinct time windows for each data split. Cross-validation techniques were employed to assess generalization, ensuring reliable evaluation of the model on unseen speech samples.

**Table 4.** Comparison of HMM and APSL–BPNN–HMM performance across five corpora.

Corpus	Model	Accuracy	Precision	Recall	F1-score
Corpus 1	HMM	0.3800	0.8250	0.2200	0.3474
	APSL–BPNN–HMM	0.7250	0.7654	0.9133	0.8328
Corpus 2	HMM	0.4150	0.7143	0.3667	0.4846
	APSL–BPNN–HMM	0.7150	0.7514	0.9267	0.8299
Corpus 3	HMM	0.4650	0.7590	0.4200	0.5408
	APSL–BPNN–HMM	0.7200	0.7640	0.9067	0.8293
Corpus 4	HMM	0.4450	0.7600	0.3800	0.5067
	APSL–BPNN–HMM	0.7500	0.7500	1.0000	0.8571
Corpus 5	HMM	0.4500	0.7381	0.4133	0.5299
	APSL–BPNN–HMM	0.7000	0.7586	0.8800	0.8148

## 7. Discussion

The proposed system translates acoustic features into language models, showing promise for effective speech recognition. However, certain phonemes, such as in “geographical” and “transmission,” were misidentified due to errors in mapping acoustic features, leading to syllable and spelling mistakes. Performance is influenced by diverse speaking styles and speaker-listener dynamics—including formal, informal, fearful, threatening, and intimate modes—which interact with psychological aspects of speech. The model adapts to unseen data, while corpus size affects memory requirements: larger dictionaries demand more resources, smaller ones are more efficient. Pronunciation variations in common names and dialect differences, such as US vs. UK standards, add complexity. Latency ranges from 3–5 seconds for typical inputs and 8–10 seconds for complex files, with a word error rate of 10%, indicating efficient recognition of out-of-corpus words. The ASPL-BPNN-HMM approach enhances phoneme identification and sequence mapping but faces challenges. Its complexity requires substantial computational power and hyperparameter tuning, including feature weights and network depth. Noise interference can degrade speech clarity, especially when background sounds mimic key phonemes. Balancing improved recognition with real-time latency remains critical. Despite these issues, ASPL shows strong potential when combined with noise reduction and optimized hyperparameters. Future enhancements include developing models using linguistic features with LSTM for faster text conversion, testing resilience to white Gaussian noise, expanding the database with diverse speaking styles, tuning HMM parameters (states, window size, cepstral coefficients), and evaluating performance on multiple languages to broaden applicability.

## 8. Conclusion

The realm of phonetics delves beyond mere phonemes, symbols, and sound utterances. It lays the crucial groundwork for mastering phonetic skills by intertwining sounds and characters. This proficiency serves as a springboard for exploring a diverse array of linguistic theories and applications, including speech recognition, speech synthesis, and discourse language transmission. In this context, the current research paper has been a testament to the empirical realization of a speech-to-text model, representing a significant stride in the field of speech recognition. The proposed methodologies have been meticulously executed on the simulation platform, Praat. The implementation unfolds across various pivotal stages, spanning from speech acquisition to feature extraction. Of note is the experimental elucidation of speech-pause detection, accomplished through an energy-based approach, as well as the feature extraction process employing framing and a window-based method embedded within the Hidden Markov Model (HMM) framework. The outcomes of these experiments have been rigorously scrutinized through established performance metrics, affirming that the acoustic modeling employed in the speech-to-text process attains an impressive level of efficacy through the utilization of HMM. This research paves the way for advanced developments in the realm of speech recognition and showcases the potential of harnessing acoustic modeling techniques for robust and efficient speech-to-text transformation.

## Data Availability

The datasets used in this research are publicly available and can be accessed from the following sources: the British National Corpus (BNC), ^27^ the American National Corpus (ANC), ^28^ the Corpus of Contemporary American English (COCA), ^29^ the Buckeye Speech Corpus, ^30^ and the Emu Speech Database. ^31^ The trained model files and derived artefacts generated during the current study are not publicly hosted due to storage and maintenance constraints. However, these materials can be made available for academic and non-commercial research purposes upon reasonable request. Any additional in-house developed datasets and the model developed in this study are available from the corresponding author upon reasonable request. Interested readers and reviewers may apply for access by contacting the corresponding author at r.siddalingappa@yorksj.ac.uk. Access will be granted subject to intended use being consistent with academic research and applicable data usage agreements.
