# Supplementary material for: Adaptive Phoneme State Learning Architecture for Enhanced Speech Recognition Using Backpropagation Neural Network and Hidden Markov Model
Source: F1000Res. 2026 Jun 3;15:338. Originally published 2026 Mar 2. [Version 2] doi: 10.12688/f1000research.177414.2 (PMC13287984; doi:10.12688/f1000research.177414.2)
Supplement: Peer review history

# Reviewer report: Reviewer response for version 2

**DOI:** 10.5256/f1000research.202186.r490689

**Published:** 2026-07-20

**Authors:** Ramanda Rizky

**Affiliations:** 1 Universitas Lancang Kuning, Pekanbaru, Riau, Indonesia

I have thoroughly evaluated the revised manuscript and the authors’ responses to the issues raised in the previous round of peer review. The revisions comprehensively address the main concerns I identified in my previous assessment. Specifically, the manuscript has been substantially strengthened through a more rigorous and up-to-date literature review, a formal mathematical derivation of the Adaptive Phoneme State Learning (APSL) algorithm, an expanded benchmark comparison with contemporary ASR approaches, and a significantly improved description of the experimental methodology and implementation details.

 The included parameter validation, speaker-level data partitioning strategies, hardware specifications, and ablation studies on the trade-off between memory and accuracy have enhanced both the reproducibility and technical credibility of the proposed framework. Furthermore, the discussion now more properly situates this contribution within the broader context of current speech recognition research, while providing a clearer justification for the proposed hybrid architecture.

 Based on these revisions, I am convinced that the authors have adequately addressed the various substantive issues regarding methodology, theory, and reproducibility that were identified during the previous review process. This manuscript has achieved a level of scientific rigor appropriate for indexing. Therefore, as I have no further comments or recommendations, I deem this manuscript suitable for acceptance in its current form.

Is the work clearly and accurately presented and does it cite the current literature?

Partly

If applicable, is the statistical analysis and its interpretation appropriate?

I cannot comment. A qualified statistician is required.

Are all the source data underlying the results available to ensure full reproducibility?

Partly

Is the study design appropriate and is the work technically sound?

Partly

Are the conclusions drawn adequately supported by the results?

Partly

Are sufficient details of methods and analysis provided to allow replication by others?

Yes

Reviewer Expertise:

English as a Foreign Language, English Language Teaching, Applied Linguistics.

I confirm that I have read this submission and believe that I have an appropriate level of expertise to confirm that it is of an acceptable scientific standard.

# Reviewer report: Reviewer response for version 2

**DOI:** 10.5256/f1000research.202186.r490687

**Published:** 2026-06-22

**Authors:** Hamza Kheddar

**Affiliations:** 1 University of Medea,, Medea, Algeria

The paper has been improved.

Is the work clearly and accurately presented and does it cite the current literature?

Yes

If applicable, is the statistical analysis and its interpretation appropriate?

Partly

Are all the source data underlying the results available to ensure full reproducibility?

No

Is the study design appropriate and is the work technically sound?

Partly

Are the conclusions drawn adequately supported by the results?

Yes

Are sufficient details of methods and analysis provided to allow replication by others?

Partly

Reviewer Expertise:

The paper has been improved

I confirm that I have read this submission and believe that I have an appropriate level of expertise to confirm that it is of an acceptable scientific standard.

# Reviewer report: Reviewer response for version 1

**DOI:** 10.5256/f1000research.195635.r479270

**Published:** 2026-05-06

**Authors:** Ramanda Rizky

**Affiliations:** 1 Universitas Lancang Kuning, Pekanbaru, Riau, Indonesia

This paper presents a hybrid Automatic Speech Recognition (ASR) framework that combines a Backpropagation Neural Network (BPNN) with a Hidden Markov Model (HMM), enhanced by an Adaptive Phoneme State Learning (APSL) mechanism. In terms of structure, this study is organized according to conventional scientific standards, proceeding systematically from preprocessing and feature extraction to modeling and evaluation. This structure supports readability and demonstrates a coherent research workflow. However, a critical review reveals several fundamental issues that limit the scientific robustness of this study, particularly regarding the relevance of benchmarks, theoretical foundation, reproducibility, and the validity of its conclusions.

 The primary concern lies in the benchmarking strategy. Although this study demonstrates that the proposed APSL-BPNN-HMM model outperforms conventional HMM baselines on metrics such as precision, recall, F1 score, and Word Error Rate, this comparison is insufficient in the context of contemporary ASR research. The field has undergone a paradigm shift toward deep learning and end-to-end architectures, including Transformer-based models, Connectionist Temporal Classification (CTC), and Recurrent Neural Network Transducers (RNN-T). By limiting the evaluation to traditional HMM baselines and, unusually, human transcriptions under noisy conditions, this study does not provide a meaningful frame of reference for assessing its contributions. Consequently, the claimed performance improvements lack external validity. To be scientifically valid, this study must include comparisons with modern ASR systems and position its contributions relative to current state-of-the-art approaches.

 Similarly important are issues of theoretical rigor. The APSL mechanism is introduced as the core innovation of this study, yet its formulation is largely procedural rather than analytical. Although the paper provides equations and step-by-step descriptions, the APSL mechanism is not clearly situated within a well-defined probabilistic or machine learning framework. The adaptive adjustment of phoneme transition probabilities using neural confidence scores appears heuristic, with limited justification grounded in established theory. This weakens both the interpretability and generalizability of the approach. A scientifically valid contribution requires not only functional implementation but also a clear theoretical foundation explaining why and under what conditions the method should work. Reinforcing this aspect involves formal derivations, clearer assumptions, and explicit connections to existing probabilistic adaptation or hybrid modeling techniques.

 Moreover, the reproducibility represents another critical limitation. Although this study describes a general workflow and reports some hyperparameters, it does not provide sufficient detail to allow for full replication. Key aspects of the experimental setup remain unclear, including the exact composition of the training, validation, and test splits; the proportions and configuration of the augmented data; and the specific preprocessing applied to each dataset. Furthermore, the absence of publicly available code, trained models, or configuration files further limits reproducibility. While the use of publicly accessible corpora is a positive step, it is insufficient on its own. In contemporary empirical research, reproducibility is closely tied to transparency, and this generally requires open access to implementation resources. Addressing this issue would significantly enhance the credibility and impact of this work.

 Dealing with data availability in this study is also incomplete. Although this study utilizes established corpora such as BNC, ANC, and COCA, the integration of these datasets with expanded data and internally generated data is not fully documented. Without clear documentation on how these datasets were combined, preprocessed, and balanced, it will be difficult for other researchers to replicate the experimental conditions or verify the reported results. Providing a detailed data protocol, including preprocessing scripts and augmentation procedures, would help bridge this gap and align this research with best practices in open and reproducible science.

 Another important limitation concerns the interpretation of results and the strength of conclusions. The findings consistently show that the proposed model outperforms the baseline HMM, which supports the internal validity of this study. However, the conclusions drawn go beyond what the available evidence can support. Claims implying performance approaching human levels or broader application to real-world ASR scenarios are not adequately supported, particularly given the lack of comparison with modern systems and the absence of statistical validation. Most of the analysis is descriptive, relying on average performance metrics without reporting variance, confidence intervals, or significance tests. This makes it difficult to determine whether the observed improvements are robust or merely coincidental. For conclusions to be scientifically justified, they must be more aligned with the scope and limitations of the experimental design.

 Broadly speaking, these issues highlight four core areas that must be addressed for this study to achieve scientific validity. First, the benchmarking framework must be expanded to include contemporary ASR models, ensuring that performance claims are evaluated against relevant standards. Second, the APSL mechanism requires a stronger theoretical foundation, moving beyond heuristic descriptions toward formal justification. Third, the study must improve reproducibility by providing detailed methodological documentation and, ideally, open access to code and data processing workflows. Fourth, conclusions should be moderated and supported by more rigorous analysis, including appropriate validation techniques. Addressing these areas will not only strengthen the internal coherence of the study but also enhance its relevance, credibility, and contribution to the evolving field of speech recognition research.

Is the work clearly and accurately presented and does it cite the current literature?

Partly

If applicable, is the statistical analysis and its interpretation appropriate?

I cannot comment. A qualified statistician is required.

Are all the source data underlying the results available to ensure full reproducibility?

Partly

Is the study design appropriate and is the work technically sound?

Partly

Are the conclusions drawn adequately supported by the results?

Partly

Are sufficient details of methods and analysis provided to allow replication by others?

Yes

Reviewer Expertise:

English as a Foreign Language, English Language Teaching, Applied Linguistics.

I confirm that I have read this submission and believe that I have an appropriate level of expertise to confirm that it is of an acceptable scientific standard.

# Reviewer report: Reviewer response for version 1

**DOI:** 10.5256/f1000research.195635.r474867

**Published:** 2026-05-06

**Authors:** Ilyos Khujayorov, Fayzulla Nazarov

**Affiliations:** 1 Artificial Intelligence, Samarkand State University named after Sharof Rashidov (Ringgold ID: 187914), Samarkand, Samarkand Province, Uzbekistan; 2 Artificial Intelligence, Tashkent University of Information Technologies named after Muhammad al-Khwarizm (Ringgold ID: 187932), Tashkent, Tashkent Province, Uzbekistan

The article proposes the APSL algorithm combining traditional BPNN and HMM models.

 The following deficiencies and suggestions were identified during the review:

1. The introduction part started with basic information related to explaining general definitions and concepts. The introduction part of scientific articles must give information like Research Context motivation, research Aims, Contributions, and others. In short, the modern problem (Problem Statement) and relevance are not revealed.
2. In the literature review part, mainly sources from 15-20 years ago are presented as main researches, but this does not reflect the current state of this field. It is appropriate to emphasize articles published within the last 3-5 years (modern State-of-the-Art models are left out of attention). Authors should completely revise the literature review, it is recommended to add publications between the years 2022–2026, especially to scientifically justify the difference between End-to-End models (Whisper, Conformer) and HMM, and to clearly show the place of the article in the era of these technologies.
3. There is no information about the hardware used in Training process.
4. How many states were used for each phoneme (for example, standard 3-state HMM or otherwise) is not clearly stated in the methodology part. This is one of the most important parameters of acoustic modeling.
5. The dynamic windowing (Eq. 18) process is not clarified. A proposal is given to increase the window size by +5 ms when the confidence coefficient is low. Theoretical or experimental bases are not presented about why it is exactly 5 ms and how this affects the time delay.
6. It is stated that the APSL mechanism reduced memory from 24 GB to 15.12 GB. However, an analysis proving that such a reduction did not negatively affect accuracy should be given in more detail in the methodology part.
7. Comparison works of the model results proposed by the authors with modern E2E architectures have not been done. This is considered one of the important issues of checking the reliability of the model. Authors must justify why they chose exactly the HMM-BPNN hybrid compared to Whisper or other modern models.
8. The mathematical expression of the APSL algorithm (17) has a heuristic appearance and its theoretical basis is not sufficiently revealed.
9. The conclusion part of the article is written in a very general way. It is appropriate to separately note the most important numerical indicators achieved in the conclusion, the role of the APSL algorithm in saving memory, and also add thoughts regarding real-time requirements.
  Due to the serious technical and methodological deficiencies noted above, I recommend rejecting this article for publication.

Is the work clearly and accurately presented and does it cite the current literature?

Yes

If applicable, is the statistical analysis and its interpretation appropriate?

Partly

Are all the source data underlying the results available to ensure full reproducibility?

No

Is the study design appropriate and is the work technically sound?

Partly

Are the conclusions drawn adequately supported by the results?

Partly

Are sufficient details of methods and analysis provided to allow replication by others?

Partly

Reviewer Expertise:

Digital signal processing,  NLP, speech recognition and synthesis,AI, parallel computing

We confirm that we have read this submission and believe that we have an appropriate level of expertise to state that we do not consider it to be of an acceptable scientific standard, for reasons outlined above.

# Response

**Published:** 2026-05-20

**Authors:** Rashmi Siddalingappa

**Affiliations:** Computer and Data Science, York St John University, York, London, UK

Comment 1

**Reviewer Comment:** The introduction part started with basic information related to explaining general definitions and concepts. The introduction part of scientific articles must give information like Research Context motivation, research Aims, Contributions, and others. In short, the modern problem (Problem Statement) and relevance are not revealed.

**Author Response:** We thank the reviewer for this important structural feedback. We have completely rewritten Section 1 (Introduction) to follow the standard scientific structure. The revised introduction now includes: (1) a clear Problem Statement identifying three fundamental challenges in ASR (accent variability, noise robustness, and interpretability); (2) a Research Gap section explicitly stating the question "How can we dynamically adapt HMM state transitions using neural network confidence scores?"; (3) a bulleted list of four specific contributions; and (4) a paper structure outline. The opening now focuses on research context and motivation rather than basic definitions.

**Author Actions:**

- Completely restructured Section 1 (Introduction)
- Added explicit Problem Statement subsection
- Added Research Gap with a focused research question
- Listed four specific contributions as bullet points
- Removed excessive basic definitions and focused on motivation

Comment 2

**Reviewer Comment:** In the literature review part, mainly sources from 15-20 years ago are presented as main researches, but this does not reflect the current state of this field. It is appropriate to emphasize articles published within the last 3-5 years (modern State-of-the-Art models are left out of attention). Authors should completely revise the literature review, it is recommended to add publications between the years 2022–2026, especially to scientifically justify the difference between End-to-End models (Whisper, Conformer) and HMM, and to clearly show the place of the article in the era of these technologies.

**Author Response:** We agree with the reviewer that the original literature review was overly focused on historical works. We have completely restructured Section 2 into four subsections. Section 2.1 (Historical Foundations) now briefly acknowledges the early work (500 BC to 2019) in a condensed manner. Section 2.2 (Traditional HMM-Based Systems) retains Table 1 but now explicitly notes the limitations of these approaches. Section 2.3 (Motivation for Hybrid HMM-Neural Approaches) is entirely new and discusses the continued relevance of hybrid models. Most importantly, we have added discussion of modern E2E architectures including Whisper (Radford et al., 2022), Conformer (Gulati et al., 2020), wav2vec 2.0 (Baevski et al., 2020), and Branchformer (Peng et al., 2022) in Section 2.2. We now clearly position HMM-based systems as complementary to E2E models for specific use cases requiring interpretability and low-resource training.

**Author Actions:**

- Restructured Section 2 into four subsections (2.1, 2.2, 2.3, 2.4)
- Added discussion of Whisper, Conformer, wav2vec 2.0, and Branchformer
- Condensed historical content while preserving key milestones
- Added explicit comparison between E2E and HMM approaches in Section 2.3
- Added new citations for modern architectures

Comment 3

**Reviewer Comment:** There is no information about the hardware used in Training process.

**Author Response:** We apologize for this omission. We have added a new subsection "Hardware and Software Environment" in Section 5.1 (Experimental Setup). The revised manuscript now specifies the complete hardware configuration including Intel Core i9 processor, NVIDIA GeForce RTX 4060 GPU (used exclusively for BPNN training), sufficient RAM, NVMe storage, Windows operating system, and software dependencies including Python 3.10, PyTorch 2.0, Praat 6.3, and Librosa 0.10. Total training time of 72 hours for 200 epochs is also reported.

**Author Actions:**

- Added "Hardware and Software Environment" subsection in Section 5.1
- Specified processor, GPU, RAM, storage, and operating system
- Listed software versions and dependencies
- Reported total training time

Comment 4

**Reviewer Comment:** How many states were used for each phoneme (for example, standard 3-state HMM or otherwise) is not clearly stated in the methodology part. This is one of the most important parameters of acoustic modeling.

**Author Response:** The reviewer is correct that this parameter was omitted. We have now added a clear statement in Section 4.2 (Windowing through Hidden Markov Model) specifying that each phoneme is modeled using a standard 3-state left-to-right HMM with states corresponding to onset, steady-state, and offset. The transition matrix A is initialized with zero probability for backward transitions, and we note that 5-state models were tested but showed no significant improvement (0.3% WER reduction) for 67% more parameters, so 3-state was retained.

**Author Actions:**

- Added specification of 3-state left-to-right HMM topology in Section 4.2
- Described states as onset, steady-state, and offset
- Reported ablation results comparing 3-state vs 5-state models
- Justified retention of 3-state based on performance vs complexity trade-off

Comment 5

**Reviewer Comment:** The dynamic windowing (Eq. 18) process is not clarified. A proposal is given to increase the window size by +5 ms when the confidence coefficient is low. Theoretical or experimental bases are not presented about why it is exactly 5 ms and how this affects the time delay.

**Author Response:** We thank the reviewer for identifying this lack of justification. We have now added empirical validation for the 5 ms parameter. Specifically, we conducted a grid search on a 500-utterance validation set evaluating candidate Δt values of 2, 3, 5, 8, 10, and 15 ms. The results showed that Δt=5 ms provides the optimal trade-off: 3.2% WER reduction with only 8% latency increase. Values below 5 ms yielded insufficient accuracy gains, while values above 5 ms produced diminishing returns (additional 0.4% WER reduction for 15%+ latency increase). This justification has been added immediately after Equation (18) in Section 4.4. We also note that 5 ms corresponds to approximately one-third of a typical English phoneme duration (15 ms), ensuring the window extension remains within the same phoneme boundary.

**Author Actions:**

- Added grid search validation for Δt parameter in Section 4.4
- Reported candidate values tested (2, 3, 5, 8, 10, 15 ms)
- Provided quantitative results (3.2% WER reduction, 8% latency increase)
- Justified 5 ms based on typical phoneme duration (15 ms)
- Added table of grid search results in the revised manuscript

Comment 6

**Reviewer Comment:** It is stated that the APSL mechanism reduced memory from 24 GB to 15.12 GB. However, an analysis proving that such a reduction did not negatively affect accuracy should be given in more detail in the methodology part.

**Author Response:** The reviewer raises an important point about verifying the memory-accuracy trade-off. We have added a new subsection "Validation of Memory-Accuracy Trade-off" in Section 5.1. We present an ablation study comparing four configurations: baseline HMM without APSL, APSL with full parameters (k=m), APSL with adaptive sharing (k=128), and APSL with aggressive sharing (k=64). The results show that adaptive sharing (k=128) achieves 96.0% accuracy compared to 95.8% with full parameters, meaning the memory reduction actually slightly improved accuracy due to implicit regularization. Aggressive sharing (k=64) reduced memory further to 11.80 GB but caused accuracy to drop to 92.3%. The similarity threshold τ=0.75 was optimized on validation data to balance sharing and specificity. This analysis confirms that the 32% memory reduction (24 GB to 15.12 GB) does not degrade accuracy and in fact provides a small benefit.

**Author Actions:**

- Added "Validation of Memory-Accuracy Trade-off" subsection in Section 5.1
- Presented ablation study with four configurations
- Showed accuracy improved from 95.8% to 96.0% with adaptive sharing
- Identified τ=0.75 as optimal similarity threshold
- Confirmed memory reduction does not harm accuracy

Comment 7

**Reviewer Comment:** Comparison works of the model results proposed by the authors with modern E2E architectures have not been done. This is considered one of the important issues of checking the reliability of the model. Authors must justify why they chose exactly the HMM-BPNN hybrid compared to Whisper or other modern models.

**Author Response:** We acknowledge this limitation and have addressed it comprehensively. First, we added a new Table 5 in Section 6 comparing our APSL-BPNN-HMM with Whisper-tiny across metrics including WER, accuracy, memory consumption, and inference time. Second, we added an explicit justification paragraph explaining three reasons for choosing HMM-BPNN: (1) controlled comparison - our primary claim is improvement over HMM baseline, not surpassing SOTA; (2) interpretability - clinical and forensic users require phoneme-level confidence scores; (3) reproducibility - our model can be trained on commodity hardware with 16 GB RAM without GPU. We also acknowledge that Whisper achieves lower WER on standard benchmarks but argue that our approach offers complementary strengths.

**Author Actions:**

- Added Table 5 comparing APSL-BPNN-HMM with Whisper-tiny
- Added justification paragraph in Section 6
- Provided three explicit reasons for HMM-BPNN choice
- Acknowledged limitations of direct comparison
- Positioned work as complementary rather than competitive

Comment 8

**Reviewer Comment:** The mathematical expression of the APSL algorithm (17) has a heuristic appearance and its theoretical basis is not sufficiently revealed.

**Author Response:** We agree with the reviewer that the original formulation lacked theoretical grounding. We have substantially revised Section 4.4 to provide a formal Bayesian derivation. We now show that the convex combination Phybrid(wi∣qi)=α(x)⋅PNN(pj∣x)+(1−α(x))⋅PHMM(wi∣qi)Phybrid​(wi​∣qi​)=α(x)⋅PNN​(pj​∣x)+(1−α(x))⋅PHMM​(wi​∣qi​) is equivalent to Bayesian model averaging under the assumption of Gaussian-distributed estimation errors, where the dynamic weighting factor is α(x)=σHMM2/(σNN2+σHMM2)α(x)=σHMM2​/(σNN2​+σHMM2​). When BPNN confidence is high (σNN2→0σNN2​→0), α(x) → 1; when HMM is more reliable, α(x) → 0. This formulation replaces the previous heuristic constant α with a theoretically justified confidence-weighting function.

**Author Actions:**

- Added Bayesian derivation of Equation (17) in Section 4.4
- Reformulated α(x) as dynamic function based on error variances
- Connected to Bayesian model averaging framework
- Provided interpretation of extreme cases (high confidence → α=1, low confidence → α=0)

Comment 9

**Reviewer Comment:** The conclusion part of the article is written in a very general way. It is appropriate to separately note the most important numerical indicators achieved in the conclusion, the role of the APSL algorithm in saving memory, and also add thoughts regarding real-time requirements.

**Author Response:** We have completely rewritten Section 8 (Conclusion) to be specific and quantitative. The revised conclusion now includes: (1) key performance metrics (96% accuracy, 95.7% recall, 92.95% precision, 94.53% F1); (2) memory efficiency gains (32% reduction from 24 GB to 15.12 GB) with explanation that adaptive sharing acts as implicit regularizer; (3) robustness across 7 accent groups and 5 noise conditions with WER <10%; (4) real-time limitations and optimization recommendations (RTF improves to 0.85 with beam search pruning and GPU acceleration); (5) specific future directions including multi-lingual extension, federated learning integration, and Transformer-based confidence estimation.

**Author Actions:**

- Completely rewrote Section 8 (Conclusion)
- Added specific numerical indicators (accuracy, recall, precision, F1)
- Highlighted memory reduction (24 GB → 15.12 GB, 32%)
- Added real-time optimization recommendations and RTF calculation
- Listed concrete future work directions

# Reviewer report: Reviewer response for version 1

**DOI:** 10.5256/f1000research.195635.r464331

**Published:** 2026-03-13

**Authors:** Hamza Kheddar

**Affiliations:** 1 University of Medea,, Medea, Algeria

The topic is interesting; however, the paper needs significant improvement:

 - The proposed APSL-BPNN-HMM architecture relies on classical models (BPNN and HMM) and does not sufficiently justify its advantages compared to modern deep learning ASR frameworks such as Transformer-based or end-to-end models (e.g., CTC, RNN-T). This limits the perceived novelty and relevance of the work in the current ASR research landscape.

 read and compare with the following for example:

 Deep Transfer Learning for Automatic Speech Recognition: Towards Better Generalization

 Machine learning approaches for automated detection and classification of dysarthria severity

 Noise-robust speech recognition: A comparative analysis of LSTM and CNN approaches

 A robust framework for noisy speech recognition using Frequency-Guided-Swin Transformer

 - The description of the Adaptive Phoneme State Learning (APSL) algorithm lacks rigorous mathematical formalization and theoretical justification. Several steps appear heuristic, and the derivation of the adaptive transition probabilities is not clearly justified or compared with existing probabilistic adaptation methods.

 - The experimental evaluation compares the proposed method mainly against a traditional HMM baseline and human transcription. However, the study does not include comparisons with contemporary ASR systems (e.g., deep neural acoustic models or end-to-end models), making it difficult to assess the true competitiveness of the proposed approach.

 - Although multiple speech corpora are used, the experimental protocol and data splitting strategy are not described in sufficient detail. The use of augmented data and partially overlapping corpora raises concerns about possible bias or insufficient independence between training and testing sets.

 - The paper claims scalability and real-time applicability, yet the architecture involves multiple processing stages (MFCC extraction, APSL segmentation, BPNN classification, and HMM decoding). The computational cost and latency are only briefly discussed (3–10 seconds for recognition), which may limit real-time deployment.

 - The authors acknowledge that pronunciation variations, dialect differences, and background noise can degrade performance, leading to phoneme misidentification in words such as “geographical” or “transmission.” This suggests the model may struggle with complex linguistic variability and real-world acoustic conditions.

 - Most all references are old-dated

Is the work clearly and accurately presented and does it cite the current literature?

Yes

If applicable, is the statistical analysis and its interpretation appropriate?

Partly

Are all the source data underlying the results available to ensure full reproducibility?

No

Is the study design appropriate and is the work technically sound?

Partly

Are the conclusions drawn adequately supported by the results?

Yes

Are sufficient details of methods and analysis provided to allow replication by others?

Partly

Reviewer Expertise:

The methodological contribution appears incremental rather than fundamentally novel

I confirm that I have read this submission and believe that I have an appropriate level of expertise to state that I do not consider it to be of an acceptable scientific standard, for reasons outlined above.

# Response

**Published:** 2026-05-20

**Authors:** Rashmi Siddalingappa

**Affiliations:** Computer and Data Science, York St John University, York, London, UK

Comment 1

**Reviewer Comment:** The proposed APSL-BPNN-HMM architecture relies on classical models (BPNN and HMM) and does not sufficiently justify its advantages compared to modern deep learning ASR frameworks such as Transformer-based or end-to-end models (e.g., CTC, RNN-T). This limits the perceived novelty and relevance of the work in the current ASR research landscape. The reviewer suggested reading and comparing with: Deep Transfer Learning for Automatic Speech Recognition, Machine learning approaches for automated detection and classification of dysarthria severity, Noise-robust speech recognition: A comparative analysis of LSTM and CNN approaches, and A robust framework for noisy speech recognition using Frequency-Guided-Swin Transformer.

**Author Response:** We thank the reviewer for this constructive criticism. We acknowledge that the original manuscript did not adequately position our hybrid approach within the context of modern end-to-end ASR architectures. To address this, we have restructured Section 2 (Research Background) into four subsections: Historical Foundations, Traditional HMM-Based Systems, Motivation for Hybrid Approaches, and Research Gap. In Section 2.3, we now explicitly discuss why hybrid HMM-neural models remain relevant for low-resource languages, medical/forensic applications requiring phoneme-level explainability, and edge deployment with memory constraints. We have also added a new subsection in Section 6 titled "Comparison with Modern End-to-End Architectures" including Table 5, which compares our APSL-BPNN-HMM with Whisper-tiny. Furthermore, we provide explicit justification for choosing HMM-BPNN over end-to-end models based on three criteria: controlled comparison with baseline, interpretability for clinical applications, and reproducibility on commodity hardware.

**Author Actions:**

- Restructured Section 2 with four subsections (2.1, 2.2, 2.3, 2.4)
- Added Section 2.3 explaining continued relevance of hybrid models
- Added new Table 5 comparing APSL-BPNN-HMM with Whisper-tiny
- Added justification paragraph for choosing HMM-BPNN over end-to-end models
- Cited relevant contemporary works including the suggested papers where appropriate

Comment 2

**Reviewer Comment:** The description of the Adaptive Phoneme State Learning (APSL) algorithm lacks rigorous mathematical formalization and theoretical justification. Several steps appear heuristic, and the derivation of the adaptive transition probabilities is not clearly justified or compared with existing probabilistic adaptation methods.

**Author Response:** We agree with the reviewer that the original APSL description was insufficiently formalized. To address this, we have substantially revised Section 4.4. We now provide a formal Bayesian derivation of the confidence-weighted probability fusion, showing that the convex combination Phybrid(wi∣qi)=α(x)⋅PNN(pj∣x)+(1−α(x))⋅PHMM(wi∣qi)Phybrid​(wi​∣qi​)=α(x)⋅PNN​(pj​∣x)+(1−α(x))⋅PHMM​(wi​∣qi​) is equivalent to Bayesian model averaging under the assumption of Gaussian-distributed estimation errors, where α(x)=σHMM2/(σNN2+σHMM2)α(x)=σHMM2​/(σNN2​+σHMM2​). This replaces the previous heuristic description with a theoretically grounded formulation. Additionally, we have added empirical justification for the 5 ms dynamic window adjustment via grid search on a 500-utterance validation set, evaluating candidate Δt values from 2 ms to 15 ms, with results showing that Δt=5 ms provides optimal trade-off (3.2% WER reduction with 8% latency increase).

**Author Actions:**

- Added Bayesian derivation of Equation (17) in Section 4.4
- Reformulated α(x) as a dynamic confidence-weighting function based on error variance
- Added grid search validation for Δt=5 ms parameter
- Included table of grid search results for candidate Δt values

Comment 3

**Reviewer Comment:** The experimental evaluation compares the proposed method mainly against a traditional HMM baseline and human transcription. However, the study does not include comparisons with contemporary ASR systems (e.g., deep neural acoustic models or end-to-end models), making it difficult to assess the true competitiveness of the proposed approach.

**Author Response:** The reviewer raises a valid point. While our primary contribution is improvement over the traditional HMM baseline, we recognize the need to contextualize our results relative to modern ASR systems. We have therefore added a new comparative analysis in Section 6 (Results) as Table 5, comparing our APSL-BPNN-HMM with Whisper-tiny (OpenAI, 2022) across metrics including WER, accuracy, memory consumption, and inference time. We explicitly acknowledge that Whisper achieves lower WER on standard benchmarks but argue that our approach offers complementary strengths in phoneme-level interpretability and training on commodity hardware without GPU requirements. We have also added a justification paragraph explaining the design trade-offs.

**Author Actions:**

- Added Table 5 comparing APSL-BPNN-HMM with Whisper-tiny
- Added justification paragraph explaining choice of HMM-BPNN over end-to-end models
- Acknowledged limitations of direct comparison due to different evaluation corpora
- Positioned our work as complementary to, rather than competitive with, SOTA E2E models

Comment 4

**Reviewer Comment:** Although multiple speech corpora are used, the experimental protocol and data splitting strategy are not described in sufficient detail. The use of augmented data and partially overlapping corpora raises concerns about possible bias or insufficient independence between training and testing sets.

**Author Response:** We apologize for the lack of clarity regarding our experimental protocol. To address this, we have added a new subsection "4.1.1 Data Partitioning and Leakage Prevention" that provides detailed information on the 70-15-15 split for each corpus, including exact sentence counts. Critically, we now specify that speaker-level partitioning was enforced across all corpora. For the Buckeye corpus (40 speakers), we allocated 32 speakers for training, 4 for validation, and 4 for testing, ensuring no individual speaker's voice appears in both training and testing sets. We also clarify that all augmented data (pitch shifting, time-stretching, noise addition) were generated only after partitioning to prevent artificial inflation of performance metrics.

**Author Actions:**

- Added subsection 4.1.1 "Data Partitioning and Leakage Prevention"
- Specified exact split percentages and sentence counts for each corpus
- Described speaker-level partitioning for Buckeye corpus (32/4/4 speakers)
- Clarified that augmentation was performed after partitioning
- Added cross-validation notes for hyperparameter tuning

Comment 5

**Reviewer Comment:** The paper claims scalability and real-time applicability, yet the architecture involves multiple processing stages (MFCC extraction, APSL segmentation, BPNN classification, and HMM decoding). The computational cost and latency are only briefly discussed (3–10 seconds for recognition), which may limit real-time deployment.

**Author Response:** We thank the reviewer for this important practical consideration. We have substantially expanded the real-time performance analysis in Section 7 (Discussion). We now provide a detailed breakdown of processing time across three input scenarios: clean short utterances (3-5 seconds, RTF 1.0-1.67), noisy long utterances (8-10 seconds, RTF 0.27-0.33), and streaming chunks (0.4-0.6 seconds per 250 ms chunk, RTF 1.6-2.4). We identify Viterbi decoding with complexity O(N × S²) as the primary bottleneck. We then provide three concrete optimization recommendations for deployment: reducing HMM states from 5 to 3, implementing beam search pruning with beam width 5, and GPU acceleration for BPNN inference (observed 3.2× speedup). Under these optimizations, we demonstrate that RTF improves to approximately 0.85 for typical utterances, making the system suitable for real-time deployment.

**Author Actions:**

- Expanded real-time performance analysis in Section 7
- Added processing time and RTF for three input scenarios
- Identified Viterbi decoding as primary bottleneck with complexity analysis
- Provided three optimization recommendations for deployment
- Calculated improved RTF (0.85) under recommended optimizations

Comment 6

**Reviewer Comment:** The authors acknowledge that pronunciation variations, dialect differences, and background noise can degrade performance, leading to phoneme misidentification in words such as "geographical" or "transmission." This suggests the model may struggle with complex linguistic variability and real-world acoustic conditions.

**Author Response:** We acknowledge this as an honest limitation of the current work. However, we note that the APSL framework's adaptive window adjustment directly addresses such variability by extending analysis windows for low-confidence phonemes. For words like "geographical" where /dʒ/ and /g/ confusion occurs, APSL's confidence threshold (θ=0.75) triggers extended windowing, which improved recognition by 18% in our ablation studies. We have added this clarification to Section 7. We also note that future work will incorporate grapheme-to-phoneme (G2P) models for out-of-vocabulary words and test on additional dialectal variants.

**Author Actions:**

- Added clarification in Section 7 that APSL's adaptive windowing addresses pronunciation variability
- Quantified improvement (18% recognition gain) for low-confidence phonemes
- Acknowledged limitation while demonstrating how APSL mitigates it
- Added future work direction for G2P integration
